# Immuno-Contexture and Immune Checkpoint Molecule Expression in Mismatch Repair Proficient Colorectal Carcinoma

**DOI:** 10.3390/cancers15123097

**Published:** 2023-06-07

**Authors:** Mauro Giacomelli, Matilde Monti, Diego Cesare Pezzola, Silvia Lonardi, Mattia Bugatti, Francesco Missale, Rossella Cioncada, Laura Melocchi, Viviana Giustini, Vincenzo Villanacci, Carla Baronchelli, Stefania Manenti, Luisa Imberti, Emanuele Giurisato, William Vermi

**Affiliations:** 1 Department of Pathology, ASST Spedali Civili di Brescia, 25123 Brescia, Italy; giacomellimauro@libero.it (M.G.); mattiabugatti16@gmail.com (M.B.); villanac@alice.it (V.V.); carla.baronchelli@gmail.com (C.B.);; 2 Department of Molecular and Translational Medicine, University of Brescia, 25123 Brescia, Italy; matilde.monti@unibs.it (M.M.); silvia.lona@gmail.com (S.L.); f.missale@unibs.it (F.M.); 3 Department of Surgery, Surgery Division II, ASST Spedali Civili di Brescia, 25123 Brescia, Italy; diego.pezzola@asst-spedalicivili.it; 4 Department of Head & Neck Oncology & Surgery Otorhinolaryngology, Antoni Van Leeuwenhoek—Nederlands Kanker Instituut, 1066 CX Amsterdam, The Netherlands; 5 Department of Pathology, Fondazione Poliambulanza, 25124 Brescia, Italy; laura.melocchi@gmail.com; 6 CREA Laboratory, AIL Center for Hemato-Oncologic Research, Diagnostic Department, ASST Spedali Civili di Brescia, 25123 Brescia, Italy; viviana.giustini@gmail.com; 7 Section of Microbiology, University of Brescia, 25123 Brescia, Italy; limberti@yahoo.it; 8 Department of Biotechnology Chemistry and Pharmacy, University of Siena, 53100 Siena, Italy; giurisato2@unisi.it; 9 Division of Cancer Sciences, School of Medical Sciences, Faculty of Biology, Medicine and Health, The University of Manchester, Manchester M13 9PL, UK; 10 Department of Pathology and Immunology, School of Medicine, Washington University, Saint Louis, MO 63130, USA

**Keywords:** colorectal cancer, mismatch repair, microsatellite instability, immune checkpoint, double negative T cells, tumor microenvironment, T-cell exhaustion, interferon-γ, PD-L1

## Abstract

**Simple Summary:**

Consensus Molecular Subtypes have recently been proposed based on molecular and immune landscape of colorectal carcinoma (CRC). Only mismatch repair deficient and hyper-mutated CRC (CRC^dMMR^) can obtain clinical benefits from immune checkpoint blockades; on the other hand, mismatch repair proficient CRCs (CRC^pMMR^) have very limited therapeutic options. This study establishes that CRC^pMMR^ displays an immunosuppressive microenvironment containing abundant tumor-associated macrophages (TAMs) and neutrophils, with a reduction in double negative T lymphocytes and B cells and increased exhausted tumor-infiltrating lymphocytes. Poor immunogenicity in CRC^pMMR^ is further supported by interferon gamma (IFN-γ) unresponsiveness of both tumor cells and TAMs.

**Abstract:**

Colorectal carcinoma (CRC) represents a lethal disease with heterogeneous outcomes. Only patients with mismatch repair (MMR) deficient CRC showing microsatellite instability and hyper-mutated tumors can obtain clinical benefits from current immune checkpoint blockades; on the other hand, immune- or target-based therapeutic strategies are very limited for subjects with mismatch repair proficient CRC (CRC^pMMR^). Here, we report a comprehensive typing of immune infiltrating cells in CRC^pMMR^. We also tested the expression and interferon-γ-modulation of PD-L1/CD274. Relevant findings were subsequently validated by immunohistochemistry on fixed materials. CRC^pMMR^ contain a significantly increased fraction of CD163^+^ macrophages (TAMs) expressing TREM2 and CD66^+^ neutrophils (TANs) together with decrease in CD4^−^CD8^−^CD3^+^ double negative T lymphocytes (DNTs); no differences were revealed by the analysis of conventional and plasmacytoid dendritic cell populations. A fraction of tumor-infiltrating T-cells displays an exhausted phenotype, co-expressing PD-1 and TIM-3. Remarkably, expression of PD-L1 on fresh tumor cells and TAMs was undetectable even after in vitro stimulation with interferon-γ. These findings confirm the immune suppressive microenvironment of CRC^pMMR^ characterized by dense infiltration of TAMs, occurrence of TANs, lack of DNTs, T-cell exhaustion, and interferon-γ unresponsiveness by host and tumor cells. Appropriate bypass strategies should consider these combinations of immune escape mechanisms in CRC^pMMR^.

## 1. Introduction

Colorectal carcinoma (CRC) represents a highly heterogeneous disease. Four main CRC Consensus Molecular Subtypes have been identified [1] based on clinical, molecular, and immune features. Biomarkers of clinical relevance are, however, limited to the occurrence of mutated oncogenes (KRAS, BRAF, NRAS, and ERBB2) and to the level of microsatellite instability (MSI) and mismatch repair (MMR) proficiency. In CRC, the microsatellite instability is due to mutations or silencing of DNA repair genes (MLH1, MSH2, MSH6, PMS2). The MMR deficient CRC (CRC^dMMR^) is a hypermutated, hypermethylated, immune proficient [2] subgroup characterized by a more favorable outcome [3], with immune checkpoint inhibitors representing the first line of systemic therapy. However, CRC^dMMR^ comprise only 5% of metastatic CRC [4]. Triple wild-type (KRAS, BRAF, and NRAS) CRC respond to anti-EGFR monoclonal antibodies in combination with chemotherapy [5], whereas the occurrence of BRAF p.Val600Glu mutation opens the window for a combination with BRAF inhibitors [6,7]. Finally, the recently emerged small group of CRC with ERBB2 amplification should be considered for appropriate targeting [8,9]. The fraction of MMR proficient CRC (CRC^pMMR^), with mutations in undruggable oncogenes, is likely poorly immunogenic and enriched in immunosuppressive cells [10,11,12], thus treated with standard chemotherapy.

Cancer immune contexture predicts prognosis and response to immune checkpoint inhibitors in different cancer types [13,14,15]. Moreover, the immune contexture becomes highly dynamic following immune checkpoint blockade, either in terms of infiltrating immune populations and expression of immune escape molecules [16,17]. In recent years, the landscape of the immune cells infiltrating CRC has been defined in terms of type, density, and location [12,18,19]. Features of CRC infiltrating immune cells predict prognosis and can be boosted by different therapeutic approaches [20,21]. Immune checkpoints are surface molecules known to be the natural feedback regulators of the normal immune response [22]. Immune checkpoint molecules can be expressed by tumor cells and cells of the microenvironment in most cancer types [23,24,25,26]. In CRC, preliminary comparative analysis indicates that the CRC^dMMR^ subgroup expresses higher levels of immune checkpoint molecules in comparison with CRC^pMMR^, the latter being also characterized by defective MHC expression [27]. Targeting the immune checkpoints has been approved in various cancer types [28,29,30], including CRC^MSI/dMMR^ [25]. On the other hand, CRC^MSS/pMMR^, which account for about 85% of cases, have been considered refractory to immune checkpoint inhibitors [31]. More recent research has shown some level of efficacy of immune checkpoint blockade combination in CRC^MSS/pMMR^, especially for the minor subgroup infiltrated with CD8^+^PD1^+^ T cells [32,33]. These emerging data suggest heterogeneity within the CRC^pMMR^ subtype in terms of immune microenvironment and immune checkpoints expression [34].

Based on the analysis of fresh tumor material, the present study defines the immune contexture of CRC^pMMR^ in terms of type, density, and expression of a set of immune checkpoints molecules. In comparison to non-cancerous colon mucosa (NM), CRC is significantly more infiltrated by CD163^+^ tumor-infiltrating macrophages (TAMs), including a fraction of immune suppressive TREM2^+^ macrophages; moreover, a significant reduction of CD3^+^CD4^−^CD8^−^ double negative T lymphocytes occurs. Finally, a fraction of T cells co-expressed immune checkpoint coupled molecules in the form of exhausted T cells. 

## 2. Materials and Methods

### 2.1. Patients

In total, 41 patients with clinically diagnosed CRC were recruited by Surgery Unit (ASST Spedali Civili of Brescia, Brescia, Italy) between January 2016 and November 2018. After surgical resection, fresh tissue from both CRC and NM was collected and processed for diagnostic purposes. All cases considered in this study underwent immunohistochemical study for mismatch repair proteins expression (MLH1, PMS2, MSH2, and MSH6) on fixed material. Clinical and pathological features were collected and summarized in Table 1. This prospective study was conducted in compliance with the Helsinki Declaration and with policies approved by the Ethics Board of ASST Spedali Civili di Brescia (WV immunocancer, NP 906).

### 2.2. Immunohistochemistry

Four-micron thick formalin-fixed paraffin-embedded (FFPE) sections of CRC tissue specimens were stained. Heat-mediated antigen retrieval was performed in microwave oven and endogenous peroxidase activity was quenched using 3% hydrogen peroxide diluted with methanol. After washing with TBS solution, slides were incubated for 1 h at room temperature with the primary antibodies (summarized in Supplementary Table S1) that required antigen retrieval (microwave in EDTA buffer, pH8.0)**.** The reaction was revealed by a 30 min incubation with a labeled horseradish peroxidase polymer (Envision+ Dual Link System, Dako and Novolink Polymer Detection System, Leica, Wetzlar, Germany) followed by 3.3′-diaminobenzidine as chromogen. Sections were counterstained with Mayer’s hematoxylin. 

Double sequential immunostainings were performed on four-micron thick FFPE sections from human tissue biopsies of non-cancerous colon mucosa (*n* = 3) with annexed Peyer’s patches and CRC (*n* = 9). Endogenous peroxidase activity was quenched using 0.3% hydrogen peroxide (Sigma-Aldrich, St. Louis, MO, USA) diluted with methanol (Sigma-Aldrich). TCRδ (1:50, clone H-14, Santa Cruz Biotechnology, Dallas, TX, USA) was applied as first, revealed using Novolink Polymer and developed with 3-amino-9-ethylcarbazole chromogen (AEC, Thermo Fisher Scientific, Waltham, MA, USA). The slides were counterstained with hematoxylin and cover-slipped using gelatin, and then were digitally scanned using Aperio Scanscope CS (Leica Microsystems, Wetzlar, Germany). The TCRδ stain was erased using ethanol as a destainer for 30 min, then antibody linking was eluted. Briefly, the slides were put in a 2-mercaptoethanol/SDS solution as previously described [35]. After 1 h washing and antigen retrieval, antibody anti-CD3 (1:70, clone LN10, Leica Biosystems, Milan, Italy) was applied to the sections. CD3 was revealed as described above for TCRδ, counterstained and digitalized. The CD3 stain was erased as described above for TCRδ and slides were subjected to another cycle of stripping. For the subsequent double staining (CD4 and CD8), after completing the first immune reaction using anti-CD4 (1:50, clone 4B12, Leica Biosystems) and DAB as chromogen, the second reaction, performed using anti-CD8 (1:50, clone C8/144B, Agilent Technologies, Santa Clara, CA, USA), was visualized using Mach 4 MR-AP (Biocare Medical, LLC, Pacheco, CA, USA), followed by the chromogen Ferangi Blue (Biocare Medical, LCC). The slides were counterstained and digitalized. The three digital slides of the same section were synchronized using the ImageScope tool, and images of representative areas for NM, Peyer patches and CRC were taken as snapshots (Supplementary Figure S1). The three snapshots were merged after hues adjustment using Adobe Photoshop. Three high power fields for each case were counted, corresponding to 0.05 mm^2^/field and two high-power fields for Peyer’s patches.

### 2.3. Tissue Processing and Flow Cytometry Analysis

Within 1 h from the surgical resection, tissue was subdivided into small fragments of 2–3 mm. The fragments were digested at 37 °C for 90 min in 5 mL of HBBS (Euroclone, Milan, Italy) supplemented with collagenase II at 200 U/mL (Worthington) and DNASE I at 1 mg/mL (Roche, Basel, Switzerland). The digestion was then stopped with 10 mL of cold RPMI containing 10% of FCS (EurloClone). The digested tissue was then filtered on a 70 μm cell-strainer and the cells were washed at 1200 rpm for 5 min at 5 °C. After blood cells lysis in RBC Lysis Buffer (Biolegend, San Diego, CA, USA), cells were washed and resuspended in cold PBS with 0.5% FCS. Cells were counted and aliquoted at 1 × 10^6^ cells/tube and labeled with vital dye Live/Dead Red (Invitrogen). After washing, cells were incubated with the following monoclonal antibodies, anti-epithelial cell adhesion molecule (EpCam)/FITC, CD19/PE, CD8/PE-Cy7 CD45/APC-H7, CD3/BV510 (Becton Dickinson, Franklin Lakes, NJ, USA), CD163/FITC, SlanDC/FITC, CD123/PE, CD16/PE, CD141/PE, CD56/PE-Vio770, CD1c/PE-Vio770, CD66b/APC, CD14/APC, HLA-DR/VioBlue, CD16/VioGreen, HLA-ABC/FITC (Miltenyi Biotec, Bergisch Gladbach, Germany), CD303/PE-Cy7, CD11b/PE-Cy7, and CD11c/BV510 (Biolegend). For immune checkpoints analysis, the following antibodies were used: anti-PD-L1/PE, PD1/PE-Cy7, TIM-3/PE (Becton Dickinson), TIGIT/APC (Biolegend), and LAG-3/PE (Miltenyi Biotec). For cell viability evaluation 7-AAD (Becton Dickinson) or Live/Dead Red (Thermo Fisher Scientific) were used. Finally, the samples were fixed in Lyse/Fix buffer (Becton Dickinson), washed, and resuspended in PBS. Samples were acquired on FACSCanto II cytometer. All flow cytometric data were analyzed with FlowJo software 10.0. (TreeStar Inc., Ashland, OR, USA). Cases with insufficient cytometric data due to tissue limitations were excluded. The gating strategies used in this study are shown in detail in Supplementary Figures from Figures S2–S11.

### 2.4. Isolation and Culture of Peripheral Blood Mononuclear Cells and Pleural Fluid Macrophages 

In total, 12 mL of whole blood were collected from healthy donors (HD). Blood was drawn directly into S-Monovette 2.7 mL K3E (1.6 mg EDTA/mL; Sarstedt, Nümbrecht, Germany, cat. no. 05.1167.001) gently rocked at room temperature until processing. Peripheral Blood Mononuclear cells (PBMCs) were obtained by Ficoll gradient. As staining control, enzymatic digestion was performed on PBMCs and compared to undigested PBMCs. Cells were washed and resuspended in cold PBS with 0.5% FCS. PBMCs were aliquoted at 1 × 10^6^ cells/tube and labeled as described above in Section 2.3. The gating strategies used in this study are reported in detail in Supplementary Figures from Figures S12–S16.

PBMCs were cultured in RPMI 1640 medium (Biochrom GmbH, Berlin, Germany) with 10% FBS (Biochrom GmbH) and were stimulated with IFN-γ 100 ng/mL (PeproTech, Inc., Rocky Hill, CT, USA) for 48 h.

Pleural fluids were collected and immediately used. Pleural fluid mononuclear cells were obtained by Ficoll gradient and processed, as previously described for PBMCs. Monocytes from peripheral blood and macrophages from pleural fluid were gated as detailed in Supplementary Figure S15.

### 2.5. PD-L1 Expression and Modulation via Interferon Gamma (IFN-γ) 

After surgical resection, cells suspension from tumor samples (*n* = 4) and their relative NM were generated as described above. Cells were subsequently stimulated with 100 ng/mL of IFN-γ (PeproTech) for 48 h, in 4 mL of RPMI medium containing 10% of heat-inactivated FCS, supplemented with 60 mg/L penicillin, 12.5 mg/L streptomycin, 2 mmol/L L-glutamine (Euroclone), and with 2.5% of BASE-128 (Alchimia, Padova, Italy). After stimulation, cells were washed and then PD-L1 expression on tumor cells and macrophages was analyzed by flow cytometry by labelling with specific antibody as described above.

### 2.6. RNAscope

To localize TGF-β positive cells, tissues were analyzed with RNAscope assay (Advanced Cell Diagnostics, Bio-Techne, Minenapolis, MN, USA) using RNAscope 2.5 HD Assay-RED kit. The Hs-TGFB1 probe (cat. no. 400881, ACDbio, Bio-Techne, Minenapolis, MN, USA) recognizes the nt 170–1649 of the TGF-β mRNA (reference sequence NM_000660.4). The sections from fixed human tissue blocks were treated following the manufacturer’s instructions. Briefly, freshly cut 3 mm sections were deparaffinized in xylene (cat. no. 06-1304F, Bio-Optica, Milan, Italy) and treated with the peroxidase block solution (cat. no. 322335, ACDbio, Bio-Techne) for 10 min at room temperature, followed by the retrieval solution for 15 min at 98 °C and by protease plus (ACDbio, cat. no. 322331) at 40 °C for 30 min. Hs-PPIB-C2 (ACDbio, cat. no. 313901) and dapB-C2 (ACDbio, cat. no. 310043-C2) were used as control probes. The hybridization was performed for 2 h at 40 °C. The signal was revealed using RNAscope 2.5 HD Detection Reagent and FAST RED.

### 2.7. Statistical Analysis 

Qualitative variables were described as absolute and relative frequencies; standard descriptive statistics were used for continuous variables, expressing means, medians, ranges, and standard deviations. Shapiro–Wilk test was applied, testing normality distribution of continuous variables. Correlation analysis was performed using Spearman’s rank correlation. Comparisons were tested by One-way ANOVA, Mann–Whitney, Kruskal–Wallis, or Wilcoxon Signed Rank test, as appropriate. For all tests, a two-tailed *p* value < 0.05 was considered significant. Statistical analysis was performed using GraphPad Prism software, Version 5.0 (San Diego, CA, USA).

## 3. Results

### 3.1. Clinical and Pathological Features of the CRC Cohort 

The main clinical and demographic characteristics of the 41 patients, including their subdivision based on disease clinical and pathological stages, are summarized in Table 1. At diagnosis, patients had a mean age of 75 years, ranging from 42 to 95 years, 26 (63.4%) were male and 15 (36.6%) were female. Since the large majority (34/41; 83%) displays proficient expression of Mismatch Repair, they were included in the classified CRC^pMMR^ subtype. Locoregional lymph-nodes were positive for metastasis in 16 (39%) cases. In total, 20 (49%) cases presented lesions involving the rectum, sigma, or the descending colon; the remaining 21 (51%) were tumors located into the ascending or transverse colon. Accordingly to the AJCC staging system, at time of diagnosis the vast majority of patients in this cohort was affected by an advanced disease (Table 1).

### 3.2. CRC^pMMR^ Tumor Tissue Displays a Reduced Infiltration of CD19^+^ B Lymphocytes and CD3^+^ Double Negative T Lymphocytes (DNTs)

**Figure 1:**
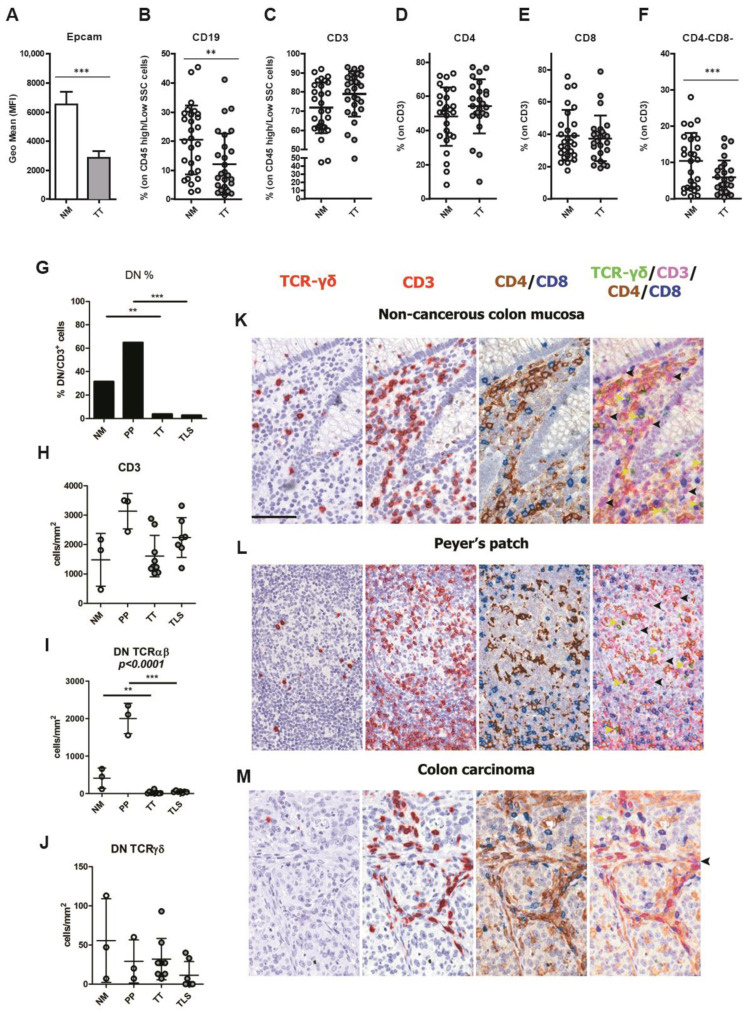
Frequency and phenotype of lymphocytes in CRC^pMMR^ tumor tissue. Expression level of EpCAM (MFI) on tumor cells is markedly reduced in TT compared to NM (**A**). Frequencies of CD19^+^ B lymphocytes (**B**), CD3^+^ T lymphocytes (**C**), CD4^+^ T helper (**D**), CD8^+^ T cytotoxic (**E**), and CD4^−^CD8^−^ double negative T lymphocytes (**F**) in NM and TT. The data (mean ± SD) are represented as histogram (**A**) or scatter dot plots (**B**–**F**). The percentage of CD4/CD8 double negative T lymphocytes (DNTs) were evaluated by immunohistochemistry on CD3^+^ T lymphocytes in non-cancerous colon mucosa (NM), Peyer’s patches (PP), colon carcinoma tumor tissues (TT), and tertiary lymphoid structures (TLS) (**G**). The cell densities of CD3^+^ T lymphocytes (**H**), TCRαβ^+^ DNTs (**I**), and TCRγδ^+^ DNTs (**J**) were evaluated by immunohistochemistry on NM, PP, TT, and TLS. The *p*-values are represented as follows; ** *p* < 0.01, *** *p* < 0.001. Immunostainings of T cells subsets on FFPE of human non-cancerous colon mucosa (**K**), including Peyer’s patches (**L**), and CRC^pMMR^ (**M**). Images are taken from digital slides stained for TCRγδ, CD3 and CD4/CD8 and overlapped after color adjustment (Supplementary Figure S1 details single steps). TCRγδ and CD3 are in red (AEC chromogen), CD4 in brown (DAB) and CD8 in blue (Ferangi Blue). Black arrows indicate CD3^+^TCRγδ^−^CD4^−^CD8^−^ cells (corresponding to TCRαβ^+^ DN) and yellow arrows indicate CD3^+^TCRγδ^+^CD4^−^CD8^−^ cells (TCRγδ^+^ DN). Numerous DN cells are found in NM, particularly in Peyer’s patches, whereas CRC^pMMR^ tumor tissue is largely devoid in this population. Magnification 400×. Scale bar: 69 µm.

Gut-associated lymphoid tissue is represented by intraepithelial lymphocytes, submucosal lymphoid cells, mesenteric lymph-nodes, and Peyer’s patches [36,37]. By using multiparametric flow cytometry, we performed phenotypical characterization of tumor-infiltrating immune cells in the tumor tissue (designated as TT; *n* = 29) compared to NM. Among the 29 TT, 25 belong to CRC^pMMR^ cases, whereas four belong to CRC^dMMR^ cases. Tumor cells were identified as EpCam^+^ cells by flow cytometry approach. It should be noted that, as previously reported [38,39], we could detect a significant reduction on EpCam expression level in TT of CRC compared to NM (*p* < 0.0001; Figure 1A).

To test the effects of the enzymatic digestion in our system, we performed the same treatment on human tonsils and PBMCs obtained from healthy donors. As reported by flow cytometry comparative analysis, enzymatic procedures did not affect the results (Supplementary Figures S7–S16). By using the Wilcoxon signed rank test, we found a significant reduction of CD19^+^ B lymphocytes (Figure 1B) in TT as compared with NM (12.08 ± 10.52 and 20.41 ± 11.84, respectively, *p* = 0.0011). The main immune cell population was represented by CD3^+^ T cells in both TT and NM. We did not observe any significant difference in CD3^+^ T lymphocyte density between NM and TT (mean 71.81% and 78.91%, respectively; Figure 1C), mostly represented by CD4^+^ T subset (NM: 48.2 ± 17.11 vs. TT: 54.23 ± 15.83, Figure 1D), followed by CD8^+^ T cells (NM: 39.24 ± 15.72 vs. TT: 37.42 ± 14.14; Figure 1E). However, a significant reduction of CD4^−^/CD8^−^ T cells was observed in TT in comparison to NM (NM: 10.47 ± 7.62 vs. TT: 5.96 ± 4.71, *p* = 0.0008; Figure 1F). Double-negative T cells express the alpha-beta or gamma-delta T-cell receptor (TCR) but lack CD4 and CD8 co-receptors. TCR-αβ^+^ or TCR-γδ^+^ double negative T lymphocytes have different immune functions [40].

To expand this finding, we analyzed three NM samples rich in double negative T lymphocytes using sequential immunostaining and identified a significant fraction of CD3^+^ DNTs (mean 466.6 cells/mm^2^, 31.4% of CD3^+^ T cells; Figure 1G,H,K and Supplementary Figure S1A) as mainly composed by TCRαβ^+^ (mean 411.1 cells/mm^2^, 27.7% of CD3^+^ T cells; Figure 1I,K), based on their negativity for TCRγδ. In NM, double negative T cells were localized in the lamina propria and in Peyer’s patches (PP, Supplementary Figure S17), but were also clearly recognizable in the B-cell follicles, where their percentage was significantly high (mean 2031/mm^2^, 64.8% of CD3^+^ T cells; Figure 1G,L and Supplementary Figure S1B). This observation is in keeping with previous findings reporting colonic DNTs [41]. We subsequently analyzed a set of human CRC (*n* = 9) and found a dramatic decrease in the CD4^−^/CD8^−^ T cells fraction (mean 61.5 cells/mm^2^, 3.8% of CD3^+^ T cells, *p* = 0.0011; Figure 1G,M and Supplementary Figure S1C) also in the context of CRC-associated tertiary lymphoid structures (TLS, Supplementary Figure S17) (*n* = 6; mean 60 cells/mm^2^, 2.7% of CD3^+^ T cells; *p* < 0.0001; Figure 1G).

### 3.3. CRC^pMMR^ Tissue Contains TREM2^+^ Tumor-Associated Macrophages (TAMs) and Neutrophils (TANs)

**Figure 2:**
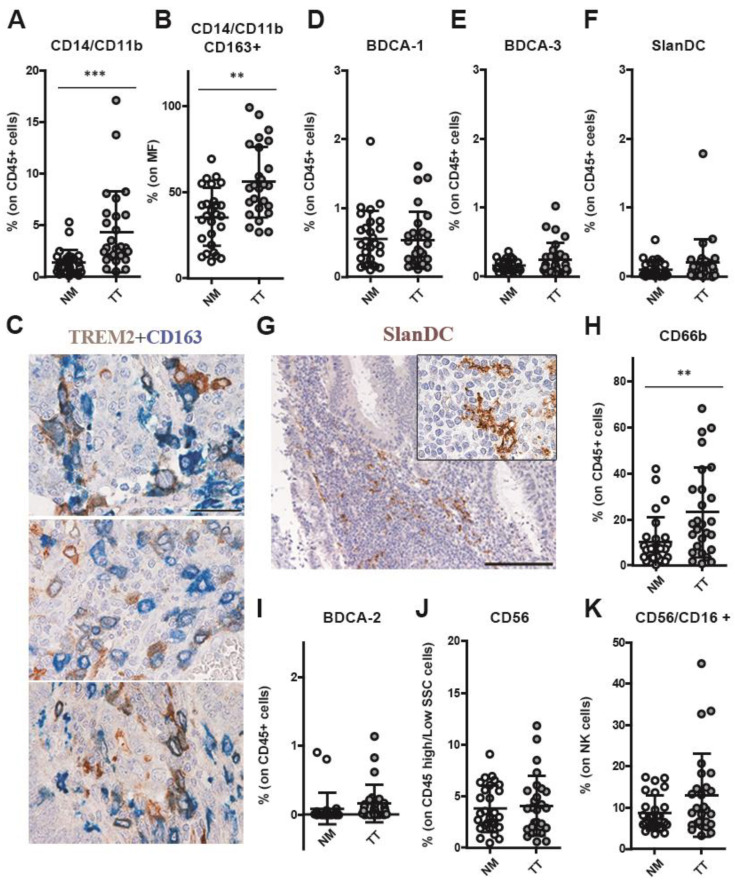
Increased density of tumor-associated macrophages and tumor-associated neutrophils in CRC^pMMR^ tumor tissue. The frequencies of CD14^+^CD11b^+^ mono/macrophages (MF; (**A**)), CD163^+^ macrophages (**B**), BDCA-1/cDC1^+^ dendritic cells (**D**), BDCA-3/CD141^+^ dendritic cells (**C**), Slan DC (**F**), CD66b^+^ granulocytes (**H**), BDCA-2/CD303^+^ plasmacytoid dendritic cells (**I**), CD56^+^ natural killer cells (NKs; (**J**)) and CD16^+^ NK cells (**K**) were evaluated by flow cytometry in CRC tumors (TT; grey) and non-cancerous colon mucosa (NM; white). The data (mean ± SD) are represented as scatter dot plots (**A**,**B**,**D**–**F**,**H**–**K**). *p* values are represented as follows; ** *p* < 0.01, *** *p* < 0.001. TREM2 expression was evaluated on CD163^+^ macrophages by double immunohistochemistry analysis (**C**). TLS localization of SlanDC was assessed by immunohistochemistry analysis (**G**). Representative sections of CRC^pMMR^ tumor biopsies are stained as labeled (**C**,**G**). Magnification 100×; scale bar: 200 µm.

We tested the innate immune compartment including dendritic cells (DC), macrophages, and polymorphonuclear leukocytes. Among myeloid cells, a significantly high frequency of macrophages, identified based on their expression of monocytes markers CD11b (integrin alpha M chain) and CD14 (endotoxin coreceptor), was observed in neoplastic tissue (3.879% ± 3.57) compared with NM (1.13% ± 1.11) (*p* < 0.0001; Figure 2A). In particular, the fraction of TAMs expressing the scavenger receptor CD163, expressed on most subpopulations of mature tissue macrophages, was highly represented in TT (56.0 ± 20.50) in comparison with NM (35.5 ± 16.85) (*p* = 0.0015; Figure 2B). Subsequently, we explored the expression of TREM2, a marker of immunosuppressive macrophages [42], in TAMs-rich CRC (*n* = 5). Double staining for CD163 and TREM2 revealed a population of TREM2^+^ macrophages distributed in the tumor stroma and within tumor nests (Figure 2C). In addition, no difference between TT and NM was observed for CD1c/BDCA-1^+^ DC and CD141/BDCA-3^+^ DC (0.53% ± 0.41 and 0.25% ± 0.24, respectively; Figure 2D,E) or for inflammatory dendritic cells that are characterized by the specific expression of the carbohydrate 6-sulfo LacNAc (SlanDC; TT: 0.20 ± 0.34 vs. NM 0.10 ± 0.12; Figure 2F), with the latter population likely restricted to TLS (Figure 2G). In contrast, we found that tumor-associated neutrophils, expressing the carcinoembryonic antigen-related glycoprotein CD66b, were present at significantly higher levels in TT as compared with NM (TT: 23.2 ± 19.6 vs. NM: 10.36 ± 10.78, *p* = 0.0017; Figure 2H). The BDCA-2/CD303^+^ plasmacytoid DC compartment was negligible in most of CRC samples (Figure 2I) with no difference between TT and NM (TT: 0.16 ± 0.27 vs. NM: 0.08 ± 0.22). Natural killer (NK) cell subset was present at low level in TT (4.03 ± 2.9) and 90.7% of these cells were CD56^+^CD16^−^ cells indicating immature NK cells and cytokine producers [43,44], but without any significant difference as compared to NM (3.80 ± 2.24; Figure 2J,K).

### 3.4. Increased Combined Expression of PD-1 and TIM-3 in CRC^pMMR^ Tumor-Infiltrating T Cells (TILs)

**Figure 3:**
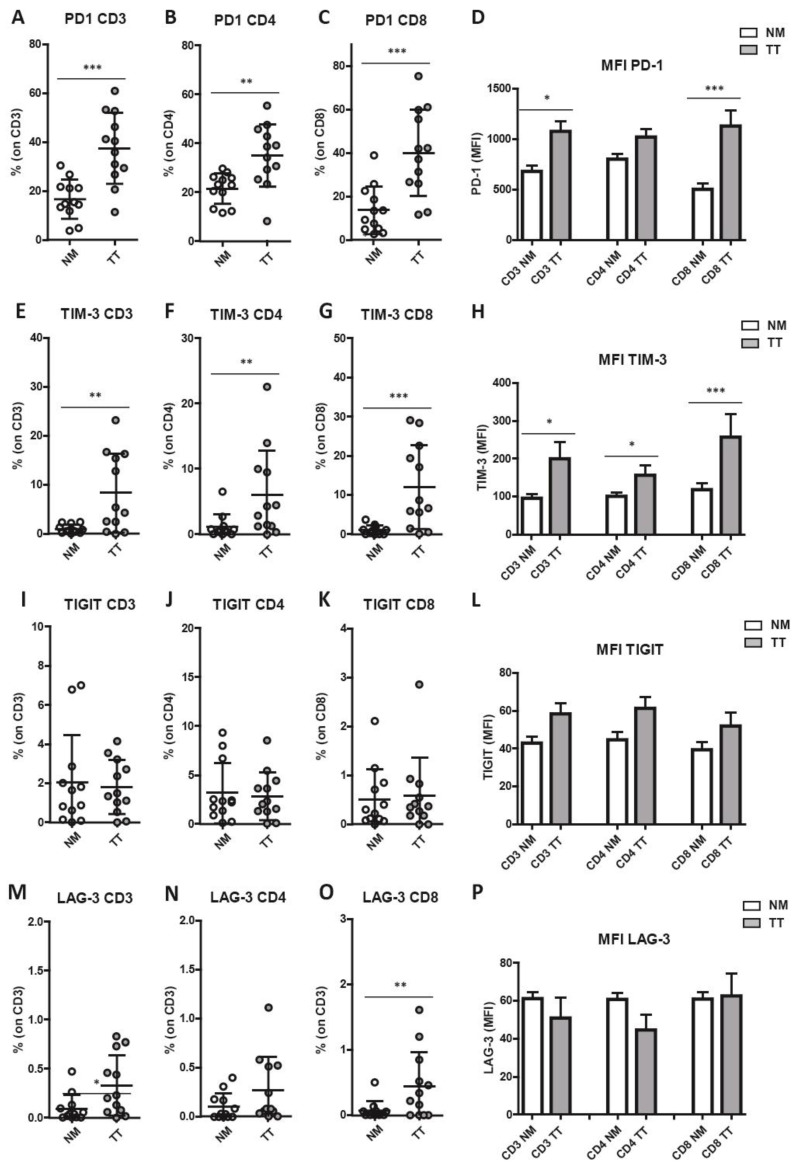
PD-1^+^ and TIM-3^+^ T lymphocytes are increased in CRC^pMMR^ samples. Immune checkpoints expression analysis reveals an enrichment of PD-1^+^ T lymphocytes within the tumor tissue (**A**–**C**) and an increased PD-1 expression level (MFI) by T lymphocytes infiltrating the tumor tissue (TT) compared to lymphocytes into non-cancerous colon mucosa (NM) (**D**). The frequency of TIM-3^+^ T lymphocytes is also increased in TT as compared to NM (**E**–**G**), as well as the TIM-3 expression level (MFI) by tumor tissue-infiltrating T lymphocytes (**H**). Distribution of TIGIT^+^ T lymphocytes (**I**–**K**) and TIGIT expression level (MFI) by tumor tissue-infiltrating T lymphocytes (**L**) shows no differences between TT and NM. Frequency of LAG-3^+^ T lymphocytes is significantly increased on CD3^+^ (**M**) and CD8^+^ T cells (**O**) in TT versus NM, whereas LAG-3 expression level (MFI) on T lymphocytes shows no significant difference between TT and NM (**P**). The percentages (**A**–**C**,**E**–**G**,**I**–**K**,**M**–**O**) and MFI (**D**,**H**,**L**,**P**) data (mean ± SD) are represented as scatter dot plots and histograms, respectively. *p* values are represented as follows; * *p* < 0.05, ** *p* < 0.01, *** *p* < 0.001.

It has been reported that immune checkpoints play important co-inhibitory/stimulatory functions on immune cells infiltrating cancer tissue [45]. Flow cytometry analysis revealed a significant increase in the percentage of T lymphocytes expressing the checkpoint receptors PD-1 (programmed cell death protein 1) and TIM-3 (T cell immunoglobulin and mucin domain-containing protein 3) and an augmented expression of PD-1 on CD8^+^ T-cells subset in TT as compared with NM (Figure 3A–D). Specifically, the frequency of PD-1^+^ T lymphocytes was significantly higher in TT as compared with NM (TT: 37.55 ± 14.52 vs. NM: 16.72 ± 8.01, *p* = 0.0010; Figure 3A). This trend was observed in both CD4^+^ T cells (TT: 35.06 ± 12.73 vs. NM: 21.45 ± 6.23, *p* = 0.0053; Figure 3B) and CD8^+^ T cells (TT: 39.97 ± 19.86 vs. NM: 13.70 ± 10.96, *p* = 0.0005; Figure 3C). In addition, PD-1 expression level on T cells was increased on TT compared with NM (for CD8^+^ subset TT: 1131 ± 539.9 vs. NM: 500.6 ± 203.1, *p* < 0.0001; Figure 3D).

**Figure 4:**
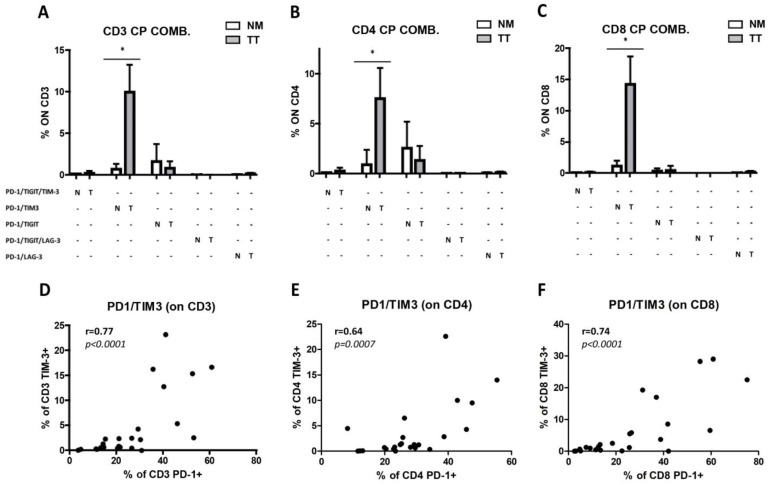
Infiltration of PD-1^+^/TIM-3^+^ T cells in CRC^pMMR^ samples. The frequency of PD-1^+^, TIM-3^+^, LAG-3^+^ and TIGIT^+^ cells were analyzed on CD3^+^ T lymphocytes (**A**), CD4^+^ T lymphocytes (**B**), and CD8^+^ T lymphocytes (**C**). Among co-expressing cells, a consistent percentage is represented by PD-1^+^TIM-3^+^ T cells in tumor samples (TT) as compared to non-cancerous colon mucosa (NM). Spearman correlation analysis between PD-1^+^ and TIM-3^+^ T cell frequencies indicates a strong correlation in the expression of both immune checkpoint molecules on both CD4^+^ and CD8^+^ T cell subsets (**D**–**F**). *p* values are represented as follows; * *p* < 0.05.

Similarly, the percentage of TIM-3^+^ T cells was significantly higher in TT as compared with NM (TT: 8.32 ± 8.05 vs. NM: 0.98 ± 0.84, *p* = 0.0015; Figure 3E–H), in both CD4^+^ T cells (TT: 6.0 ± 6.85 vs. NM: 1.19 ± 1.82, *p* = 0.0024; Figure 3F) and CD8^+^ T cells (TT: 12.03 ± 10.68 vs. NM: 1.06 ± 1.16, *p* = 0.0010; Figure 3G). Similarly, TIM-3 expression level on T lymphocytes was increased (Figure 3H) in CD3^+^ T cells (TT: 198.9 ± 153.3 vs. NM: 94.8 ± 38.5, *p* = 0.012), CD4^+^ T cells (TT: 155.1 ± 91.7 vs. NM: 99.35 ± 35.7, *p* = 0.0122) and CD8^+^ T-cells subsets (TT: 257.3 ± 211.3 vs. NM: 117.4 ± 59.28, *p* = 0.0024). A very low percentage and significant difference between TT and NM was observed for T lymphocytes expressed the immune checkpoint T cell immunoreceptor with Ig and ITIM domains (TIGIT) (Figure 3I–L). Additionally, the percentage of T lymphocytes expressing lymphocyte activation gene 3 protein (LAG-3) was very low on CD3^+^ (TT: 0.33 ± 0.30 vs. NM: 0.09 ± 0.14, *p* = 0.018; Figure 3M), CD4^+^ (TT: 0.27 ± 0.34 vs. NM: 0.10 ± 0.13; ns; Figure 3N), and CD8^+^ T-cells subsets (TT: 0.45 ± 0.52 vs. NM: 0.07 ± 0.14, *p* = 0.009; Figure 3O) of both TT and NM samples and no increased LAG-3 expression level was found in TT (Figure 3P). Boolean analysis of combined immune checkpoint molecules expression on T lymphocytes showed that PD-1^+^/TIM-3^+^ double positive cells were the major subset represented in TT (Figure 4A–C). Indeed, we observed a significant increase in PD-1^+^TIM-3^+^ T cells in TT as compared with NM (*p* = 0.014 in CD3^+^, *p* = 0.014 in CD8^+^, and *p* = 0.028 in CD4^+^). Among PD-1^+^TIM-3^+^ T lymphocytes, Spearman analysis revealed a strong correlation of the expression of these two immune checkpoints molecules on CD3^+^ T lymphocytes (*p* < 0.0001, R = 0.77; Figure 4D). A similar correlation was observed on CD4^+^ (*p* = 0.0007, R = 0.64; Figure 4F) and on CD8^+^ T lymphocytes (*p* < 0.0001, R = 0.74; Figure 4F). No additional correlations were found between the other immune checkpoints evaluated (*p* > 0.05). 

### 3.5. Defective Modulation of PD-L1 by IFN-γ on CRC^pMMR^ Tumor Cells and Tumor-Associated Macrophages (TAMs)

**Figure 5:**
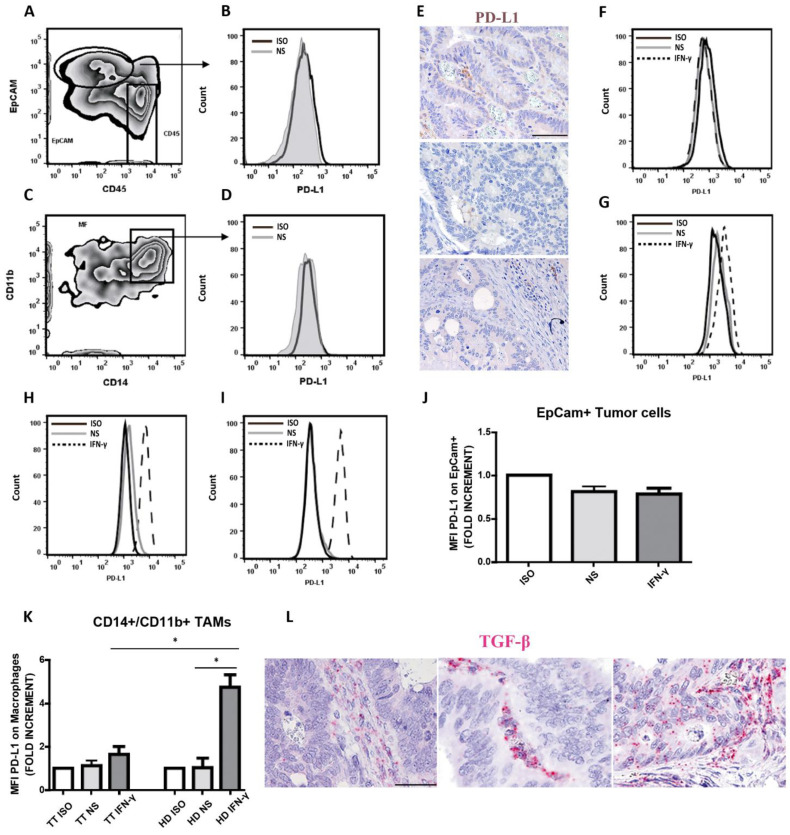
Lack of PD-L1 expression on CRC^pMMR^-derived tumor cells and tumor-associated macrophages. A representative case showing lack of PD-L1 expression on CRC^pMMR^-derived Epcam^+^ tumor cells (**A**,**B**) and CD14^+^CD11b^+^ TAMs (**C**,**D**) by flow cytometry. Histograms illustrate fluorescence intensity of PD-L1 expression at base line (unstimulated, NS) and isotype control (ISO) (**B**,**D**) on gated cells (**A**,**C**), as indicated by arrows. Lack of PD-L1 expression was confirmed by immunohistochemistry in most tumor cells and TAMs (**E**). Magnification 200×; scale bar: 100 µm. Representative PD-L1 expression on CRC^pMMR^-derived Epcam^+^ tumor cells (**F**), CRC^pMMR^-derived CD14^+^CD11b^+^ macrophages (**G**), peripheral blood monocytes from healthy donors (HD; (**H**)) and macrophages from pleural fluid (**I**). Histograms show the fluorescence intensity of isotype controls (ISO) and PD-L1 on both unstimulated (NS) and IFN-γ stimulated single cell suspensions (**F**–**I**). Analysis of PD-L1 expression (MFI) on EpCam^+^ tumor cells shows lack of PD-L1 modulation after 48 h of IFN-γ stimulation (*n* = 4; (**J**)). Analysis of PD-L1 expression (MFI) on CRC^pMMR^-derived CD14^+^CD11b^+^ macrophages (indicated as TT; *n* = 4) and peripheral blood monocytes from HD (*n* = 3) shows that PD-L1 is strongly induced in IFN-γ stimulated HDs monocytes, whereas CRC^pMMR^-derived macrophages poorly respond to IFN-γ stimulation (**K**). Histogram plots represent mean value ± SD of fold increment normalized to isotype control. *p* values are represented as follows; * *p* < 0.05 (**J**,**K**). TGF-β mRNA expression was evaluated on CRC tumor biopsies using RNAscope. Three representative sections showing TGF-β positive stromal and tumor cells are reported (**L**). Magnification 200×; scale bar: 100 µm.

We could not detect any expression of the immune checkpoint molecule PD-1 ligand (PD-L1) on tumor cells (Figure 5A,B). Moreover, PD-L1 expression on TAMs was barely detectable also by immunohistochemistry (Figure 5C–E). Lack of detection of PD-L1 expression might derive from a limited availability of local interferon or other known stimuli [46,47]. Alternatively, lack of PD-L1 expression might be due to reduced sensitivity to IFN-γ by tumor and immune cells. To test this hypothesis, Epcam^+^ carcinoma cells and TAMs obtained after tissue digestion of selected CRC^pMMR^ cases (*n* = 4) were cultured and stimulated with IFN-γ for 48 h. All these four cases resulted negative for PD-L1, as documented by flow cytometry at the base line and we did not observe any expression of PD-L1 on tumor cells, even after IFN-γ stimulation, which suggests that CRC^pMMR^ are largely unresponsive to IFN-γ (Figure 5F,J). 

Similarly, very low expression of PD-L1 was observed in CRC^pMMR^-infiltrating CD11b^+^CD14^+^ TAMs both unstimulated and stimulated with IFN-γ (Figure 5G,K). In contrast, we found a strong induction of PD-L1 expression on circulating monocytes from healthy donors (Figure 5H,K) and pleural fluid macrophages (Figure 5I), taken as positive controls. Since sensitivity to PD-L1 modulating stimuli might depend on immunosuppressive cytokines including TGF-β, we measured these molecules by using RNAscope. Accordingly, TGF-β transcript were detected in all four tested cases and mainly localized in the tumor stroma (Figure 5L).

## 4. Discussion

By comparative analysis with NM, this work dissected the immune cell composition of TT of CRC in which the pMMR subtype was largely prevalent. We could observe a significant increase in tumor-associated macrophages and neutrophils in TT, together with a reduction of CD3^+^ DNTs, the latter mainly composed by TCRαβ^+^ T cells, as previously reported for other cancer types [48,49,50]. The relevance of this finding relies on the potential anti-tumor function of CD4^−^CD8^−^ T lymphocytes. These cells represent a small, but heterogeneous, subset of mature T lymphocytes, containing both TCRαβ^+^ and TCRγδ^+^ T cells [51]. TCRαβ^+^ DNTs are widely distributed among tissues and blood and can develop from thymic progenitors [52,53], as well as from peripheral CD8^+^ [54,55] and CD4^+^ precursors [56]. The role of double negative T cells has been explored in cancer revealing either antitumor or tumor-supportive activity depending on the tumor type [51]. Human DNTs were first developed as adoptive cell therapy endowed with antitumor and cytotoxic activity against acute myeloid leukemia, pancreatic cancer, and non-small cells lung cancer [57,58,58,59,60]. Based on our findings, a better characterization of double negative T lymphocytes in CRC might define their role as an adoptive cell therapy in CRC^pMMR^.

The immunoscore [61] is one of the strongest prognosticators in CRC, regardless of the MSI status [19,62,62,63]. Large studies performed analyzing CRC^MSS/pMMR^ patients showed a prognostic relevance of Th1 cells and cytotoxic immune infiltration in human CRC [64,65]. B lymphocytes represent a major component of CRC-infiltrating immune cells, predominant at the invasive margins [18,65]. In this study, we observed a significant reduction in B lymphocytes in CRC^pMMR^ TT as compared to the relative NM, most of which likely derive from TLS or their precursors. The occurrence of TLS plays an active role in the organization of the local adaptive immune response against malignant cells; furthermore, TLS are associated with favorable outcome in various cancer types, including localized and metastatic CRC [66,67,68,69]. Finally, in immune checkpoint blockade-treated patients, the enrichment of B cells and TLS is associated with significant therapeutic advantages [70].

Among innate immune cells, tumor-associated macrophages and neutrophils resulted significantly increased in TT. Macrophages have been mainly reported as tumor-promoting cells in many types of tumors [71,72,73,74], through their ability to produce immunosuppressive cytokines, such as interleukin (IL)-10 and IL-1Ra [75]. TAMs are also known to promote tumor angiogenesis, tissue remodeling, and inhibition of the immune response producing IL-10, TGF-β and Indoleamine 2,3-dioxygenase (IDO) instead of IL-12. These later trajectories lead to the development of regulatory T cells and effector T cells anergy [75]. The density of CD163^+^ macrophages is associated with poor prognosis and reduced overall survival in CRC, in addition to many other solid tumors [71,72,73,74]. However, recently identified markers have shown extreme heterogeneity among TAM population [42,76,77,78]. The presence of TREM2^+^CD163^+^ macrophages suggests an immunosuppressive and tumor promoting phenotype of CRC^pMMR^ TAMs, as previously reported [79]. 

Recently, the expression of immune checkpoints, such as PD-L1 or V-domain immunoglobulin suppressor of T-cell activation (VISTA), has been described on TAMs, particularly after treatment with Ipilimumab, revealing a dynamic escape mechanism after immune checkpoint inhibitors [16]. The expression of these immune checkpoints was evident on two distinct macrophage subsets, and the subset with VISTA expression was often positive also for CD163 and ARG1, highlighting a subset of inhibitory macrophages [16,19] that makes this macrophages subpopulation a potential target for the development of future anticancer therapies [16,33,80]. Our study fails to detect expression of PD-L1 on CRC^pMMR^ tumor cells and tumor-associated macrophages, thus confirming previous observations [81] of uncommon expression of PD-L1 on tumor cells (only 2% of their cases) or macrophages. Specifically, PD-L1 expression is restricted to the invasive front of CRC^dMMR^ or limited to CRC^pMMR^ cases with high lymphocytes infiltrate. These data point out a correlation between PD-L1 expression and high levels of TILs in human CRC. Other studies [82] indicate that PD-L1 expression correlates with the density of CD8^+^ TILs in CRC^dMMR^, suggesting ongoing adaptive immune resistance. Our in vitro study suggests that CRC^pMMR^ cancer cells can be insensitive to IFN-γ and might also mediate their inhibitory effect on TAM. In fact, in our experimental conditions, IFN-γ stimulation was conducted in the simultaneous presence of tumor cells and immune cells. TGF-β has the ability to downregulate the expression of PD-L1 on monocytes [83], also by counteracting the IFN-γ effects on gene expression, through the MEK/ERK kinase pathway [84]. Therefore, the secretion of TGF-β by CRC cells could partially explain the poor PD-L1 induction by IFN-γ observed on whole cell suspensions of CRCs. A large fraction of CRCs have an altered TGF-β pathway and are characterized by a high production of TGF-β [85], which is associated with a higher frequency of relapse and a worse outcome. Accordingly, we could detect TGF-β transcript, mainly localized in the tumor stroma of CRC^pMMR^. However, based on the limited CRC cases tested in this study, further confirmatory investigations are required.

Although highly heterogeneous, the tumor-associated neutrophils density was significantly increased in TT compared to NM. Many studies have clarified that the plasticity between the anti-tumorigenic or tumor-promoting phenotype of neutrophils can escape therapeutic intervention [86,87]. Although neutrophils were originally considered as effector cells, it has been largely documented that tumor-associated neutrophils promote angiogenesis and tumor cell dissemination to distant sites [88]. Specifically, TANs have been demonstrated to boost liver metastasis of CRC [89]. Moreover, the neutrophil-to-lymphocyte ratio is a well-defined predictive biomarker for CRC patients [90,91]. The clinical significance of CD66b^+^ neutrophils in the invasive margins of CRC patients depends also on the co-occurrence of CD8^+^ TILs [92]. However, the phenotype and function of neutrophils in CRC subtypes have been scarcely investigated. The existing immune checkpoint inhibitors are effective in CRC^MSI/dMMR^ patients but had little effect in CRC^MSS/pMMR^. However, CRC^MSS/pMMR^ represent most CRC cases. Therefore, new strategies to improve the efficacy of immune checkpoint blockade in these patients are needed. As an important component of the microenvironment in CRC [93], tumor-associated neutrophils might overturn the current immunotherapeutic approach suggesting that blockade of neutrophils expansion and functions could be employed as antitumor therapeutic strategies [94]. 

We observed a significant increase in both the frequency of PD-1^+^ and TIM3^+^ TILs and their PD-1 and TIM-3 expression level in CRC TT versus NM. On the contrary, TIGIT and LAG-3 expression were detected at low levels in CRC^pMMR^, and on a small fraction of TILs. In CRC, PD-1 was shown to be upregulated on exhausted CD8^+^ T cells [95]. Based on our set of data, CRC^pMMR^ is infiltrated by TIM-3^+^PD-1^+^ T cells, a phenotype associated with a significant decreased T-cell activity [96]. 

Finally, as a side finding of our flow cytometry strategy, we observed a significant reduction in EpCam expression on tumor cells. Recently, a multivariate analysis of EpCam expression in CRC by immunohistochemistry has demonstrated that its downregulation represents an independent prognostic factor associated with poor disease-specific survival [38] and is associated with an increase in the migratory capacity of the tumor cells [97,98]. EpCam is a key molecule for homophilic cell to cell adhesion [38,39,99,100] and is involved in tumor cell proliferation and adhesion via cadherin [101,102]. In addition, EGF-mediated stimulation cleaves the cytoplasmic domain of EpCam (EpICD) and leads to the internalization and nuclear localization of EpCID, resulting in the transcription of genes involved in migration, adhesion, and epithelial-mesenchymal transition in endometrial cancer [103]. EpCID also induces the activation of β-catenin pathway [104,105]. 

## 5. Conclusions

Overall, this study highlights immune features of CRC^pMMR^ on fresh tumor tissue. The main limitation of the current study is represented by lack of comparative analysis with a significant CRC^dMMR^ group, which based on their expected frequency, require a larger prospective recruitment. However, present data and literature comparisons support a more limited immunogenicity CRC^pMMR^ and identify novel windows of therapeutic opportunity by overcoming escape mechanisms other than that PD1/PD-L1.

## Figures and Tables

**Table 1:** Demographics and clinicopathological features of CRC patients cohort.

Pt ^1^	Gender	Age	Anatomic District ^2^	Istotype ^3^	G ^4^	N ^5^	Neoplastic Emboli ^6^	Infl. CT ^7^	Infl. IM ^8^	Crohn-like ^9^	MMR ^10^	TNM	AJCC
#1	M	42	R	SR		N1	L1	+	+	no	P	pT3N2b	IIIc
#2	M	74	L	NOS	HIGH	N1	V1 + L1	+	+	no	P	pT4aN2a	IIIc
#3	M	43	R	NOS	LOW	N0	L1	+	+	no	P	pT3N0	IIa
#4	M	70	L	NOS	LOW	N1	L1	++	++	yes	P	pT4aN1b	IIIb
#5	M	76	L	NOS	LOW	N1	V1 + L1	+	+	no	P	pT4aN2bM1a	IVa
#6	F	77	L	NOS	LOW	N0	L1	++	++	yes	P	pT3N0	IIa
#7	M	76	R	NOS	LOW	N1	L1	+	+	no	P	pT3N1b	IIIb
#8	M	79	L	NOS	LOW	N0	V1	+	+	no	P	pT3N0	IIa
#9	M	60	L	NOS	LOW	N1	L1	+	+	no	P	pT3N1b	IIIb
#10	F	59	L	NOS	LOW	N1	V0-L0	+	+	yes	P	pT4aN2b	IIIc
#11	M	69	R	MUC	LOW	N0	V1	+	+	yes	P	pT3N1c	IIIb
#12	F	80	R	NOS	LOW	N0	V1 + L1	+	+	no	P	pT3N0	IIa
#13	F	84	R	MUC	HIGH	N0	V0-L0	+	+	yes	D	pT3N0	IIa
#14	M	66	R	NOS	LOW	N0	V0-L0	+	+	yes	P	pT3N0	IIa
#15	M	73	L	MUC	LOW	N1	L1	+	+	no	P	pT3N1a	IIIb
#16	M	77	R	MUC	LOW	N0	L1	+	+	no	P	pT3N0	IIa
#17	F	81	L	MUC	LOW	N0	L1	+++	+++	no	P	pT4bN0	IIc
#18	F	59	L	NOS	LOW	N0	V0-L0	+	+	yes	P	pT3N0	IIa
#19	F	75	R	NOS	LOW	N0	L1	+	+	no	P	pT3N0	IIa
#20	F	46	L	NOS	LOW	N0	L1	+	+	no	P	pT2N0	I
#21	M	78	L	SR	LOW	N0	L1	-	-	no	P	pT2N0	I
#22	F	79	R	MUC	LOW	N0	L1	+	+	no	D	pT3N0 (pT2N0)	IIa (I)
#23	M	77	R	NOS	LOW	N1	V1 + L1	+	+	no	P	pT3N1b	IIIb
#24	M	72	L	NOS	LOW	N0	V0-L0	+	+	no	P	pT2N0	I
#25	F	52	L	NOS	LOW	N1	V1 + L1	+	+	no	P	pT4aN2b	IIIc
#26	M	80	R	NOS	LOW	N0	L1	+	+	yes	P	pT4bN0	IIb
#27	M	95	L	NOS	LOW	N0	L1	+	+	no	P	pT3N0	IIa
#28	F	47	R	NOS	LOW	N0	L1	+	+	no	D	pT3N0	IIa
#29	M	88	R	SR	LOW	N0	V1 + L1	+	+	no	D	pT4aN0	IIb
#30	F	71	R	NOS	HIGH	N0	L1	+	+	no	D	pT4aN1c	IIIb
#31	F	92	R	NOS	LOW	N1	L1	+	-	no	P	pT4aN1a	IIIb
#32	M	46	R	NOS	HIGH	N0	L1	-	+	yes	P	pT4aN0	IIb
#33	M	76	R	NOS	LOW	N0	L1	-	-	no	D	pT3N0	IIa
#34	F	70	L	NOS	LOW	N1	V1 + L1	-	-	no	P	pT3N2a	IIIb
#35	M	78	L	NOS	LOW	N0	V0-L0	-	+	yes	P	pT1N0	I
#36	M	58	L	NOS	LOW	N1	V1 + L1	+	+	no	P	pT3N1b	IIIb
#37	M	75	R	NOS	LOW	N1	L1	+	+	yes	P	pT3N2a	IIIb
#38	M	80	L	NOS	LOW	N1	L1	-	-	yes	P	pT3N1b	IIIb
#39	F	71	R	MUC	LOW	N0	L1	-	-	no	D	pT3n0	IIa
#40	M	79	L	NOS	LOW	N1	V1 + L1	+	+	no	P	pT4aN1b	IIIb
#41	M	79	R	NOS	LOW	N0	V0-L0	+	+++	no	P	pT3n0	IIa

^1^ Patients; ^2^ Anatomic district R = ileum, ascending or transverse colon; L = rectum, sigma or descending colon; ^3^ Istotype MUC = mucinous, SG = signet ring, NOS = not otherwise specified; ^4^ Grading LOW = G1-G2; HIGH = G3 and undifferentiated; empty space = data unknown; ^5^ Lymph node metastasis N0 = absent, N1 = present; ^6^ Neoplastic Emboli L1 = lymphatic vessels, V1 = venous vessels, L0-V0 = no evidence; ^7^ Inflam CT = Inflammation in the center of tumor; ^8^ Inflam IM = Inflammation at the invasive margin; ^9^ Crohn-like = inflammation Crohn-like, yes = feature present, no = feature not present; ^10^ MMR = mismatch repair, P = proficient, D = deficient.

## Data Availability

All data generated or analyzed during this study are included in this published article and its supplementary information files.

## Supplementary Materials

The following supporting information can be downloaded at: https://www.mdpi.com/article/10.3390/cancers15123097/s1, Figure S1: Identification of DN T cells in normal colon and CRC: adjustment steps to final merge; Figure S2: Representative gating strategy for the identification of B lymphocytes, granulocytes, and NK lymphocytes subsets in colon mucosae; Figure S3: Representative gating strategy for the identification of pDC subset in colon mucosae; Figure S4: Representative gating strategy for the identification of T lymphocytes subset in colon mucosae; Figure S5: Representative gating strategy for the identification of macrophage subset in colon mucosae; Figure S6: Representative gating strategy for the identification of myeloid cell subsets in colon mucosae; Figure S7: Representative gating strategy for the identification of granulocytes, B lymphocytes and NK cells subsets in tonsils; Figure S8: Representative gating strategy for the identification of pDC subset in tonsil; Figure S9: Representative gating strategy for the identification of T lymphocytes subset in tonsil; Figure S10: Representative gating strategy for the identification of macrophage subset in tonsil; Figure S11: Representative gating strategy for the identification of myeloid cell subsets in tonsil; Figure S12: Immunophenotype of B lymphocytes, granulocytes and NK lymphocytes from enzymatically digested PBMCs in comparison with undigested PBMCs; Figure S13: Immunophenotype of pDCs from enzymatically digested PBMCs in comparison with undigested PBMCs; Figure S14: Immunophenotype of T lymphocytes from enzymatically digested PBMCs in comparison with undigested PBMCs; Figure S15: Immunophenotype of macrophages from enzymatically digested PBMCs in comparison with undigested PBMCs; Figure S16: Immunophenotype of myeloid cells from enzymatically digested PBMCs in comparison with undigested PBMCs; Figure S17: Histology of the Peyer’s Patches (PP) in non-cancerous colon mucosa and Tertiary Lymphoid Structures (TLS) in CRC tumor tissues. Table S1: List of antibodies used for IHC staining.

## References

[1] Guinney J., Dienstmann R., Wang X., de Reyniès A., Schlicker A., Soneson C., Marisa L., Roepman P., Nyamundanda G., Angelino P. (2015). The consensus molecular subtypes of colorectal cancer. Nat. Med..

[2] Picard E., Verschoor C.P., Ma G.W., Pawelec G. (2020). Relationships between immune landscapes, genetic subtypes and responses to immunotherapy in colorectal cancer. Front. Immunol..

[3] Kang S., Na Y., Joung S.Y., Lee S.I., Oh S.C., Min B.W. (2018). The significance of microsatellite instability in colorectal cancer after controlling for clinicopathological factors. Medicine.

[4] Biller L.H., Schrag D. (2021). Diagnosis and treatment of metastatic colorectal cancer: A review. JAMA.

[5] Lo Nigro C., Ricci V., Vivenza D., Granetto C., Fabozzi T., Miraglio E., Merlano M.C. (2016). Prognostic and predictive biomarkers in metastatic colorectal cancer anti-EGFR therapy. World J. Gastroenterol..

[6] Kopetz S., Grothey A., Yaeger R., Van Cutsem E., Desai J., Yoshino T., Wasan H., Ciardiello F., Loupakis F., Hong Y.S. (2019). Encorafenib, Binimetinib, and Cetuximab in BRAF V600E-Mutated Colorectal Cancer. N. Engl. J. Med..

[7] Tabernero J., Grothey A., Van Cutsem E., Yaeger R., Wasan H., Yoshino T., Desai J., Ciardiello F., Loupakis F., Hong Y.S. (2021). Encorafenib Plus Cetuximab as a New Standard of Care for Previously Treated BRAF V600E-Mutant Metastatic Colorectal Cancer: Updated Survival Results and Subgroup Analyses from the BEACON Study. J. Clin. Oncol..

[8] Meric-Bernstam F., Hurwitz H., Raghav K.P.S., McWilliams R.R., Fakih M., VanderWalde A., Swanton C., Kurzrock R., Burris H., Sweeney C. (2019). Pertuzumab plus trastuzumab for HER2-amplified metastatic colorectal cancer (MyPathway): An updated report from a multicentre, open-label, phase 2a, multiple basket study. Lancet Oncol..

[9] Sartore-Bianchi A., Trusolino L., Martino C., Bencardino K., Lonardi S., Bergamo F., Zagonel V., Leone F., Depetris I., Martinelli E. (2016). Dual-targeted therapy with trastuzumab and lapatinib in treatment-refractory, KRAS codon 12/13 wild-type, HER2-positive metastatic colorectal cancer (HERACLES): A proof-of-concept, multicentre, open-label, phase 2 trial. Lancet Oncol..

[10] Chen J., Ye X., Pitmon E., Lu M., Wan J., Jellison E.R., Adler A.J., Vella A.T., Wang K. (2019). IL-17 inhibits CXCL9/10-mediated recruitment of CD8+ cytotoxic T cells and regulatory T cells to colorectal tumors. J. Immunother. Cancer.

[11] Kamal Y., Schmit S.L., Frost H.R., Amos C.I. (2020). The tumor microenvironment of colorectal cancer metastases: Opportunities in cancer immunotherapy. Immunotherapy.

[12] Dienstmann R., Vermeulen L., Guinney J., Kopetz S., Tejpar S., Tabernero J. (2017). Consensus molecular subtypes and the evolution of precision medicine in colorectal cancer. Nat. Rev. Cancer.

[13] Hanna G.J., Liu H., Jones R.E., Bacay A.F., Lizotte P.H., Ivanova E.V., Bittinger M.A., Cavanaugh M.E., Rode A.J., Schoenfeld J.D. (2017). Defining an inflamed tumor immunophenotype in recurrent, metastatic squamous cell carcinoma of the head and neck. Oral Oncol..

[14] Chevrier S., Levine J.H., Zanotelli V.R.T., Silina K., Schulz D., Bacac M., Ries C.H., Ailles L., Jewett M.A.S., Moch H. (2017). An immune atlas of clear cell renal cell carcinoma. Cell.

[15] Lavin Y., Kobayashi S., Leader A., Amir E.-A.D., Elefant N., Bigenwald C., Remark R., Sweeney R., Becker C.D., Levine J.H. (2017). Innate Immune Landscape in Early Lung Adenocarcinoma by Paired Single-Cell Analyses. Cell.

[16] Gao J., Ward J.F., Pettaway C.A., Shi L.Z., Subudhi S.K., Vence L.M., Zhao H., Chen J., Chen H., Efstathiou E. (2017). VISTA is an inhibitory immune checkpoint that is increased after ipilimumab therapy in patients with prostate cancer. Nat. Med..

[17] Benci J.L., Xu B., Qiu Y., Wu T.J., Dada H., Twyman-Saint Victor C., Cucolo L., Lee D.S.M., Pauken K.E., Huang A.C. (2016). Tumor interferon signaling regulates a multigenic resistance program to immune checkpoint blockade. Cell.

[18] Bindea G., Mlecnik B., Tosolini M., Kirilovsky A., Waldner M., Obenauf A.C., Angell H., Fredriksen T., Lafontaine L., Berger A. (2013). Spatiotemporal dynamics of intratumoral immune cells reveal the immune landscape in human cancer. Immunity.

[19] Mlecnik B., Bindea G., Angell H.K., Maby P., Angelova M., Tougeron D., Church S.E., Lafontaine L., Fischer M., Fredriksen T. (2016). Integrative analyses of colorectal cancer show immunoscore is a stronger predictor of patient survival than microsatellite instability. Immunity.

[20] Pohl M., Schmiegel W. (2016). Therapeutic Strategies in Diseases of the Digestive Tract—2015 and Beyond Targeted Therapies in Colon Cancer Today and Tomorrow. Dig. Dis..

[21] Seow H.F., Yip W.K., Fifis T. (2016). Advances in targeted and immunobased therapies for colorectal cancer in the genomic era. Onco Targets Ther..

[22] Miko E., Meggyes M., Doba K., Barakonyi A., Szereday L. (2019). Immune checkpoint molecules in reproductive immunology. Front. Immunol..

[23] Masugi Y., Nishihara R., Yang J., Mima K., da Silva A., Shi Y., Inamura K., Cao Y., Song M., Nowak J.A. (2017). Tumour CD274 (PD-L1) expression and T cells in colorectal cancer. Gut.

[24] Hua D., Sun J., Mao Y., Chen L.-J., Wu Y.-Y., Zhang X.-G. (2012). B7-H1 expression is associated with expansion of regulatory T cells in colorectal carcinoma. World J. Gastroenterol..

[25] Llosa N.J., Cruise M., Tam A., Wicks E.C., Hechenbleikner E.M., Taube J.M., Blosser R.L., Fan H., Wang H., Luber B.S. (2015). The vigorous immune microenvironment of microsatellite instable colon cancer is balanced by multiple counter-inhibitory checkpoints. Cancer Discov..

[26] Solinas C., Garaud S., De Silva P., Boisson A., Van den Eynden G., de Wind A., Risso P., Rodrigues Vitória J., Richard F., Migliori E. (2017). Immune Checkpoint Molecules on Tumor-Infiltrating Lymphocytes and Their Association with Tertiary Lymphoid Structures in Human Breast Cancer. Front. Immunol..

[27] Toor S.M., Sasidharan Nair V., Murshed K., Abu Nada M., Elkord E. (2021). Tumor-Infiltrating Lymphoid Cells in Colorectal Cancer Patients with Varying Disease Stages and Microsatellite Instability-High/Stable Tumors. Vaccines.

[28] Angelova M., Charoentong P., Hackl H., Fischer M.L., Snajder R., Krogsdam A.M., Waldner M.J., Bindea G., Mlecnik B., Galon J. (2015). Characterization of the immunophenotypes and antigenomes of colorectal cancers reveals distinct tumor escape mechanisms and novel targets for immunotherapy. Genome Biol..

[29] Jacobs J., Smits E., Lardon F., Pauwels P., Deschoolmeester V. (2015). Immune checkpoint modulation in colorectal cancer: What’s new and what to expect. J. Immunol. Res..

[30] Xiao Y., Freeman G.J. (2015). The microsatellite instable subset of colorectal cancer is a particularly good candidate for checkpoint blockade immunotherapy. Cancer Discov..

[31] Lichtenstern C.R., Ngu R.K., Shalapour S., Karin M. (2020). Immunotherapy, inflammation and colorectal cancer. Cells.

[32] Fan A., Wang B., Wang X., Nie Y., Fan D., Zhao X., Lu Y. (2021). Immunotherapy in colorectal cancer: Current achievements and future perspective. Int. J. Biol. Sci..

[33] Makaremi S., Asadzadeh Z., Hemmat N., Baghbanzadeh A., Sgambato A., Ghorbaninezhad F., Safarpour H., Argentiero A., Brunetti O., Bernardini R. (2021). Immune checkpoint inhibitors in colorectal cancer: Challenges and future prospects. Biomedicines.

[34] El Hajj J., Reddy S., Verma N., Huang E.H., Kazmi S.M. (2023). Immune Checkpoint Inhibitors in pMMR/MSS Colorectal Cancer. J. Gastrointest. Cancer.

[35] Giurisato E., Lonardi S., Telfer B., Lussoso S., Risa-Ebrí B., Zhang J., Russo I., Wang J., Santucci A., Finegan K.G. (2020). Extracellular-Regulated Protein Kinase 5-Mediated Control of p21 Expression Promotes Macrophage Proliferation Associated with Tumor Growth and Metastasis. Cancer Res..

[36] Garside P., Millington O., Smith K.M. (2004). The anatomy of mucosal immune responses. Ann. N. Y. Acad. Sci..

[37] MacPherson G., Milling S., Yrlid U., Cousins L., Turnbull E., Huang F.-P. (2004). Uptake of antigens from the intestine by dendritic cells. Ann. N. Y. Acad. Sci..

[38] Goossens-Beumer I.J., Zeestraten E.C.M., Benard A., Christen T., Reimers M.S., Keijzer R., Sier C.F.M., Liefers G.J., Morreau H., Putter H. (2014). Clinical prognostic value of combined analysis of Aldh1, Survivin, and EpCAM expression in colorectal cancer. Br. J. Cancer.

[39] Kim J.H., Bae J.M., Song Y.S., Cho N.-Y., Lee H.S., Kang G.H. (2016). Clinicopathologic, molecular, and prognostic implications of the loss of EPCAM expression in colorectal carcinoma. Oncotarget.

[40] Velikkakam T., Gollob K.J., Dutra W.O. (2022). Double-negative T cells: Setting the stage for disease control or progression. Immunology.

[41] Carrasco A., Fernández-Bañares F., Pedrosa E., Salas A., Loras C., Rosinach M., Aceituno M., Andújar X., Forné M., Zabana Y. (2016). Regional specialisation of T cell subsets and apoptosis in the human gut mucosa: Differences between ileum and colon in healthy intestine and inflammatory bowel diseases. J. Crohns Colitis.

[42] Molgora M., Esaulova E., Vermi W., Hou J., Chen Y., Luo J., Brioschi S., Bugatti M., Omodei A.S., Ricci B. (2020). TREM2 Modulation Remodels the Tumor Myeloid Landscape Enhancing Anti-PD-1 Immunotherapy. Cell.

[43] Lajoie L., Congy-Jolivet N., Bolzec A., Thibault G. (2017). Gradual Increase of FcγRIIIa/CD16a Expression and Shift toward IFN-γ Secretion during Differentiation of CD56dim Natural Killer Cells. Front. Immunol..

[44] Messaoudene M., Fregni G., Fourmentraux-Neves E., Chanal J., Maubec E., Mazouz-Dorval S., Couturaud B., Girod A., Sastre-Garau X., Albert S. (2014). Mature cytotoxic CD56(bright)/CD16(+) natural killer cells can infiltrate lymph nodes adjacent to metastatic melanoma. Cancer Res..

[45] O’Neill R.E., Cao X. (2019). Co-stimulatory and co-inhibitory pathways in cancer immunotherapy. Adv. Cancer Res..

[46] Garcia-Diaz A., Shin D.S., Moreno B.H., Saco J., Escuin-Ordinas H., Rodriguez G.A., Zaretsky J.M., Sun L., Hugo W., Wang X. (2017). Interferon Receptor Signaling Pathways Regulating PD-L1 and PD-L2 Expression. Cell Rep..

[47] Chen S., Crabill G.A., Pritchard T.S., McMiller T.L., Wei P., Pardoll D.M., Pan F., Topalian S.L. (2019). Mechanisms regulating PD-L1 expression on tumor and immune cells. J. Immunother. Cancer.

[48] Di Blasi D., Boldanova T., Mori L., Terracciano L., Heim M.H., De Libero G. (2020). Unique T-Cell Populations Define Immune-Inflamed Hepatocellular Carcinoma. Cell. Mol. Gastroenterol. Hepatol..

[49] Stankovic B., Bjørhovde H.A.K., Skarshaug R., Aamodt H., Frafjord A., Müller E., Hammarström C., Beraki K., Bækkevold E.S., Woldbæk P.R. (2018). Immune Cell Composition in Human Non-small Cell Lung Cancer. Front. Immunol..

[50] Greenplate A.R., McClanahan D.D., Oberholtzer B.K., Doxie D.B., Roe C.E., Diggins K.E., Leelatian N., Rasmussen M.L., Kelley M.C., Gama V. (2019). Computational Immune Monitoring Reveals Abnormal Double-Negative T Cells Present across Human Tumor Types. Cancer Immunol. Res..

[51] Wu Z., Zheng Y., Sheng J., Han Y., Yang Y., Pan H., Yao J. (2022). CD3+CD4-CD8- (Double-Negative) T Cells in Inflammation, Immune Disorders and Cancer. Front. Immunol..

[52] Collin R., Lombard-Vadnais F., Hillhouse E.E., Lebel M.-È., Chabot-Roy G., Melichar H.J., Lesage S. (2020). MHC-Independent Thymic Selection of CD4 and CD8 Coreceptor Negative αβ T Cells. J. Immunol..

[53] Grandjean C.L., Sumaria N., Martin S., Pennington D.J. (2017). Increased TCR signal strength in DN thymocytes promotes development of gut TCRαβ(+)CD8αα(+) intraepithelial lymphocytes. Sci. Rep..

[54] Crispín J.C., Tsokos G.C. (2009). Human TCR-αβ^+^ CD_4_^−^ CD_8_^−^ T cells can derive from CD_8_^+^ T cells and display an inflammatory effector phenotype. J. Immunol..

[55] Rodríguez-Rodríguez N., Flores-Mendoza G., Apostolidis S.A., Rosetti F., Tsokos G.C., Crispín J.C. (2020). TCR-α/β CD4- CD8- double negative T cells arise from CD8+ T cells. J. Leukoc. Biol..

[56] Ford M.S., Zhang Z.-X., Chen W., Zhang L. (2006). Double-negative T regulatory cells can develop outside the thymus and do not mature from CD8+ T cell precursors. J. Immunol..

[57] Chen J., Hu P., Wu G., Zhou H. (2019). Antipancreatic cancer effect of DNT cells and the underlying mechanism. Pancreatology.

[58] Lu Y., Hu P., Zhou H., Yang Z., Sun Y.U., Hoffman R.M., Chen J. (2019). Double-negative T Cells Inhibit Proliferation and Invasion of Human Pancreatic Cancer Cells in Co-culture. Anticancer Res..

[59] Yao J., Ly D., Dervovic D., Fang L., Lee J.B., Kang H., Wang Y.-H., Pham N.-A., Pan H., Tsao M.-S. (2019). Human double negative T cells target lung cancer via ligand-dependent mechanisms that can be enhanced by IL-15. J. Immunother. Cancer.

[60] Gomes A.Q., Martins D.S., Silva-Santos B. (2010). Targeting γδ T lymphocytes for cancer immunotherapy: From novel mechanistic insight to clinical application. Cancer Res..

[61] Galon J., Pagès F., Marincola F.M., Thurin M., Trinchieri G., Fox B.A., Gajewski T.F., Ascierto P.A. (2012). The immune score as a new possible approach for the classification of cancer. J. Transl. Med..

[62] Pagès F., Mlecnik B., Marliot F., Bindea G., Ou F.-S., Bifulco C., Lugli A., Zlobec I., Rau T.T., Berger M.D. (2018). International validation of the consensus Immunoscore for the classification of colon cancer: A prognostic and accuracy study. Lancet.

[63] Taylor E.S., McCall J.L., Girardin A., Munro F.M., Black M.A., Kemp R.A. (2016). Functional impairment of infiltrating T cells in human colorectal cancer. Oncoimmunology.

[64] Fridman W.H., Pagès F., Sautès-Fridman C., Galon J. (2012). The immune contexture in human tumours: Impact on clinical outcome. Nat. Rev. Cancer.

[65] Ogino S., Nosho K., Irahara N., Meyerhardt J.A., Baba Y., Shima K., Glickman J.N., Ferrone C.R., Mino-Kenudson M., Tanaka N. (2009). Lymphocytic reaction to colorectal cancer is associated with longer survival, independent of lymph node count, microsatellite instability, and CpG island methylator phenotype. Clin. Cancer Res..

[66] Meshcheryakova A., Tamandl D., Bajna E., Stift J., Mittlboeck M., Svoboda M., Heiden D., Stremitzer S., Jensen-Jarolim E., Grünberger T. (2014). B cells and ectopic follicular structures: Novel players in anti-tumor programming with prognostic power for patients with metastatic colorectal cancer. PLoS ONE.

[67] Sautès-Fridman C., Lawand M., Giraldo N.A., Kaplon H., Germain C., Fridman W.H., Dieu-Nosjean M.-C. (2016). Tertiary lymphoid structures in cancers: Prognostic value, regulation, and manipulation for therapeutic intervention. Front. Immunol..

[68] Di Caro G., Bergomas F., Grizzi F., Doni A., Bianchi P., Malesci A., Laghi L., Allavena P., Mantovani A., Marchesi F. (2014). Occurrence of tertiary lymphoid tissue is associated with T-cell infiltration and predicts better prognosis in early-stage colorectal cancers. Clin. Cancer Res..

[69] Bergomas F., Grizzi F., Doni A., Pesce S., Laghi L., Allavena P., Mantovani A., Marchesi F. (2011). Tertiary intratumor lymphoid tissue in colo-rectal cancer. Cancers.

[70] Xia J., Xie Z., Niu G., Lu Z., Wang Z., Xing Y., Ren J., Hu Z., Hong R., Cao Z. (2023). Single-cell landscape and clinical outcomes of infiltrating B cells in colorectal cancer. Immunology.

[71] Shabo I., Olsson H., Sun X.-F., Svanvik J. (2009). Expression of the macrophage antigen CD163 in rectal cancer cells is associated with early local recurrence and reduced survival time. Int. J. Cancer.

[72] Shabo I., Olsson H., Elkarim R., Sun X.-F., Svanvik J. (2014). Macrophage infiltration in tumor stroma is related to tumor cell expression of CD163 in colorectal cancer. Cancer Microenviron..

[73] Shabo I., Stål O., Olsson H., Doré S., Svanvik J. (2008). Breast cancer expression of CD163, a macrophage scavenger receptor, is related to early distant recurrence and reduced patient survival. Int. J. Cancer.

[74] Klingen T.A., Chen Y., Aas H., Wik E., Akslen L.A. (2017). Tumor-associated macrophages are strongly related to vascular invasion, non-luminal subtypes, and interval breast cancer. Hum. Pathol..

[75] Sica A., Schioppa T., Mantovani A., Allavena P. (2006). Tumour-associated macrophages are a distinct M2 polarised population promoting tumour progression: Potential targets of anti-cancer therapy. Eur. J. Cancer.

[76] Nalio Ramos R., Missolo-Koussou Y., Gerber-Ferder Y., Bromley C.P., Bugatti M., Núñez N.G., Tosello Boari J., Richer W., Menger L., Denizeau J. (2022). Tissue-resident FOLR2+ macrophages associate with CD8+ T cell infiltration in human breast cancer. Cell.

[77] Bugatti M., Bergamini M., Missale F., Monti M., Ardighieri L., Pezzali I., Picinoli S., Caronni N., Missolo-Koussou Y., Helft J. (2022). A population of TIM4+FOLR2+ macrophages localized in tertiary lymphoid structures correlates to an active immune infiltrate across several cancer types. Cancer Immunol. Res..

[78] Ardighieri L., Missale F., Bugatti M., Gatta L.B., Pezzali I., Monti M., Gottardi S., Zanotti L., Bignotti E., Ravaggi A. (2021). Infiltration by CXCL10 secreting macrophages is associated with antitumor immunity and response to therapy in ovarian cancer subtypes. Front. Immunol..

[79] Cheruku S., Rao V., Pandey R., Rao Chamallamudi M., Velayutham R., Kumar N. (2023). Tumor-associated macrophages employ immunoediting mechanisms in colorectal tumor progression: Current research in Macrophage repolarization immunotherapy. Int. Immunopharmacol..

[80] Zhong X., Chen B., Yang Z. (2018). The Role of Tumor-Associated Macrophages in Colorectal Carcinoma Progression. Cell. Physiol. Biochem..

[81] Liu S., Gönen M., Stadler Z.K., Weiser M.R., Hechtman J.F., Vakiani E., Wang T., Vyas M., Joneja U., Al-Bayati M. (2019). Cellular localization of PD-L1 expression in mismatch-repair-deficient and proficient colorectal carcinomas. Mod. Pathol..

[82] Rosenbaum M.W., Bledsoe J.R., Morales-Oyarvide V., Huynh T.G., Mino-Kenudson M. (2016). PD-L1 expression in colorectal cancer is associated with microsatellite instability, BRAF mutation, medullary morphology and cytotoxic tumor-infiltrating lymphocytes. Mod. Pathol..

[83] Ou J.-N., Wiedeman A.E., Stevens A.M. (2012). TNF-α and TGF-β counter-regulate PD-L1 expression on monocytes in systemic lupus erythematosus. Sci. Rep..

[84] Park I.-K., Letterio J.J., Gorham J.D. (2007). TGF-beta 1 inhibition of IFN-gamma-induced signaling and Th1 gene expression in CD4+ T cells is Smad3 independent but MAP kinase dependent. Mol. Immunol..

[85] Calon A., Espinet E., Palomo-Ponce S., Tauriello D.V.F., Iglesias M., Céspedes M.V., Sevillano M., Nadal C., Jung P., Zhang X.H.-F. (2012). Dependency of colorectal cancer on a TGF-β-driven program in stromal cells for metastasis initiation. Cancer Cell.

[86] Jaillon S., Ponzetta A., Di Mitri D., Santoni A., Bonecchi R., Mantovani A. (2020). Neutrophil diversity and plasticity in tumour progression and therapy. Nat. Rev. Cancer.

[87] Mollinedo F. (2019). Neutrophil degranulation, plasticity, and cancer metastasis. Trends Immunol..

[88] Mizuno R., Kawada K., Itatani Y., Ogawa R., Kiyasu Y., Sakai Y. (2019). The Role of Tumor-Associated Neutrophils in Colorectal Cancer. Int. J. Mol. Sci..

[89] Hirai H., Fujishita T., Kurimoto K., Miyachi H., Kitano S., Inamoto S., Itatani Y., Saitou M., Maekawa T., Taketo M.M. (2014). CCR1-mediated accumulation of myeloid cells in the liver microenvironment promoting mouse colon cancer metastasis. Clin. Exp. Metastasis.

[90] Pedrazzani C., Mantovani G., Fernandes E., Bagante F., Luca Salvagno G., Surci N., Campagnaro T., Ruzzenente A., Danese E., Lippi G. (2017). Assessment of neutrophil-to-lymphocyte ratio, platelet-to-lymphocyte ratio and platelet count as predictors of long-term outcome after R0 resection for colorectal cancer. Sci. Rep..

[91] Halazun K.J., Aldoori A., Malik H.Z., Al-Mukhtar A., Prasad K.R., Toogood G.J., Lodge J.P.A. (2008). Elevated preoperative neutrophil to lymphocyte ratio predicts survival following hepatic resection for colorectal liver metastases. Eur. J. Surg. Oncol..

[92] Yin C., Okugawa Y., Yamamoto A., Kitajima T., Shimura T., Kawamura M., Tsujiura M., Okita Y., Ohi M., Toiyama Y. (2022). Prognostic significance of CD8+ tumor-infiltrating lymphocytes and CD66b+ tumor-associated neutrophils in the invasive margins of stages I-III colorectal cancer. Oncol. Lett..

[93] Galdiero M.R., Bianchi P., Grizzi F., Di Caro G., Basso G., Ponzetta A., Bonavita E., Barbagallo M., Tartari S., Polentarutti N. (2016). Occurrence and significance of tumor-associated neutrophils in patients with colorectal cancer. Int. J. Cancer.

[94] Zheng W., Wu J., Peng Y., Sun J., Cheng P., Huang Q. (2022). Tumor-Associated Neutrophils in Colorectal Cancer Development, Progression and Immunotherapy. Cancers.

[95] Wu X., Zhang H., Xing Q., Cui J., Li J., Li Y., Tan Y., Wang S. (2014). PD-1(+) CD8(+) T cells are exhausted in tumours and functional in draining lymph nodes of colorectal cancer patients. Br. J. Cancer.

[96] Arai Y., Saito H., Ikeguchi M. (2012). Upregulation of TIM-3 and PD-1 on CD4+ and CD8+ T Cells Associated with Dysfunction of Cell-Mediated Immunity after Colorectal Cancer Operation. Yonago Acta Med..

[97] Gosens M.J.E.M., van Kempen L.C.L., van de Velde C.J.H., van Krieken J.H.J.M., Nagtegaal I.D. (2007). Loss of membranous Ep-CAM in budding colorectal carcinoma cells. Mod. Pathol..

[98] Went P., Dirnhofer S., Salvisberg T., Amin M.B., Lim S.D., Diener P.-A., Moch H. (2005). Expression of epithelial cell adhesion molecule (EpCam) in renal epithelial tumors. Am. J. Surg. Pathol..

[99] Balzar M., Winter M.J., de Boer C.J., Litvinov S.V. (1999). The biology of the 17-1A antigen (Ep-CAM). J. Mol. Med..

[100] Van der Gun B.T.F., Melchers L.J., Ruiters M.H.J., de Leij L.F.M.H., McLaughlin P.M.J., Rots M.G. (2010). EpCAM in carcinogenesis: The good, the bad or the ugly. Carcinogenesis.

[101] Maetzel D., Denzel S., Mack B., Canis M., Went P., Benk M., Kieu C., Papior P., Baeuerle P.A., Munz M. (2009). Nuclear signalling by tumour-associated antigen EpCAM. Nat. Cell Biol..

[102] Winter M.J., Nagelkerken B., Mertens A.E.E., Rees-Bakker H.A.M., Briaire-de Bruijn I.H., Litvinov S.V. (2003). Expression of Ep-CAM shifts the state of cadherin-mediated adhesions from strong to weak. Exp. Cell Res..

[103] Hsu Y.-T., Osmulski P., Wang Y., Huang Y.-W., Liu L., Ruan J., Jin V.X., Kirma N.B., Gaczynska M.E., Huang T.H.-M. (2016). EpCAM-Regulated Transcription Exerts Influences on Nanomechanical Properties of Endometrial Cancer Cells That Promote Epithelial-to-Mesenchymal Transition. Cancer Res..

[104] Chaves-Pérez A., Mack B., Maetzel D., Kremling H., Eggert C., Harréus U., Gires O. (2013). EpCAM regulates cell cycle progression via control of cyclin D1 expression. Oncogene.

[105] Jachin S., Bae J.S., Sung J.J., Park H.S., Jang K.Y., Chung M.J., Kim D.G., Moon W.S. (2014). The role of nuclear EpICD in extrahepatic cholangiocarcinoma: Association with β-catenin. Int. J. Oncol..

