# Supplementary material for: Immuno-Contexture and Immune Checkpoint Molecule Expression in Mismatch Repair Proficient Colorectal Carcinoma
Source: Cancers (Basel). 2023 Jun 7;15(12):3097. doi: 10.3390/cancers15123097 (PMC10296282; doi:10.3390/cancers15123097)
Supplement: Supplementary file 1 [file cancers-15-03097-s001.zip › cancers-2409922-Supplementary Figures.pdf]

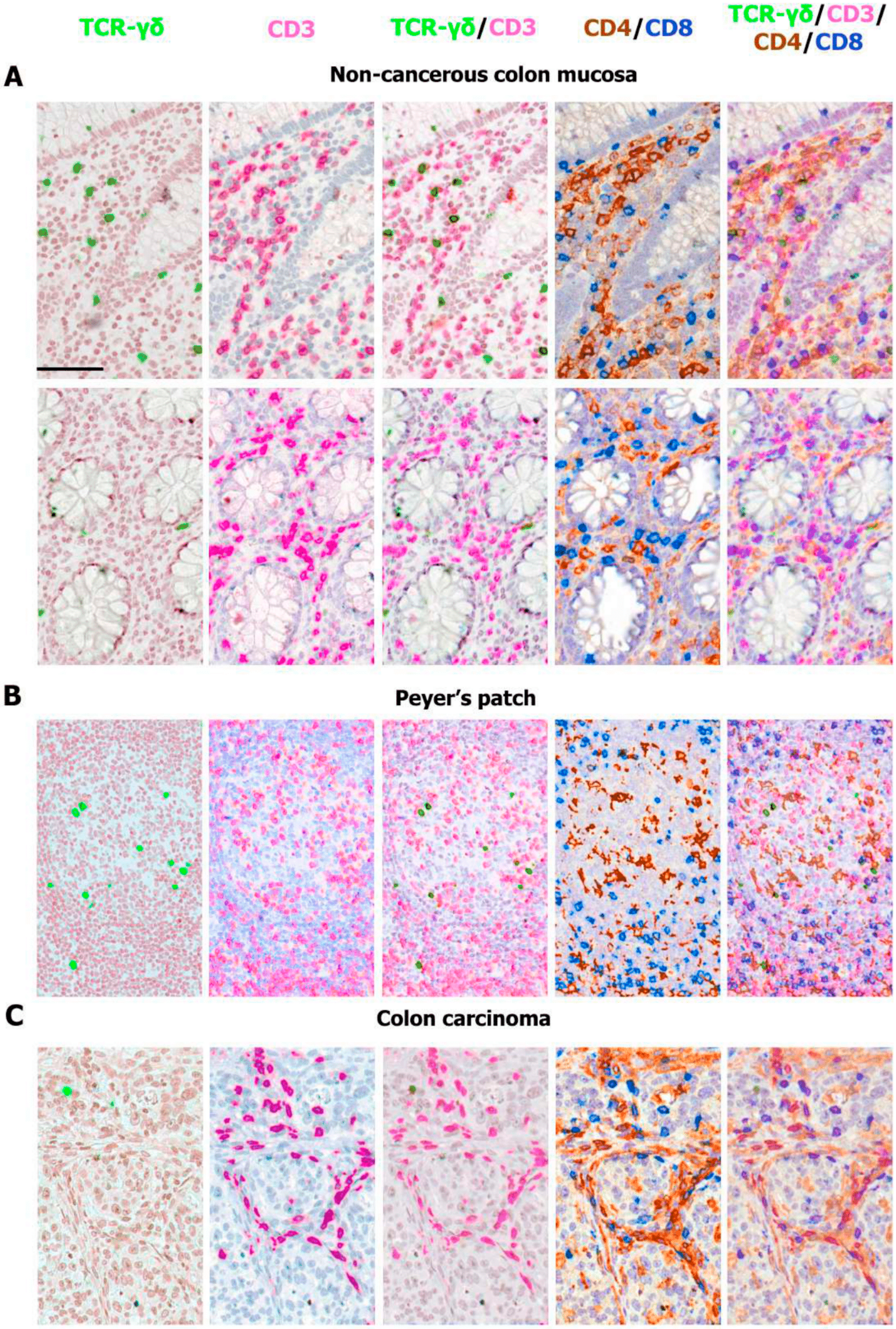

Figure S1. Identification of DN T cells in normal colon and CRC: adjustment steps to final merge. Stainings are from FFPE of human normal colon mucosa (**A**), including Peyer's patches (**B**), and colon carcinoma (**C**) biopsies stained as labeled. The original images were taken as snapshots from digital slides and adjusted changing hues and saturation for TCR and CD3 that were originally stained using the same red chromogen (they turned respectively to green and fuchsia; original images are shown in Figure 1) in order to highlight the single markers when images would have been merged. Only saturation was adjusted for CD4 (brown) and CD8 (blue). Intermediate and final merged images were also shown. Two normal colic mucosa cases with different number

of TCR + cells (**A**), a representative Peyer's patch (**B**) and a representative colon carcinoma with paucity of DN T cells (**C**) are shown. Magnification 400x. Scale bar: 69 um.

(colon)

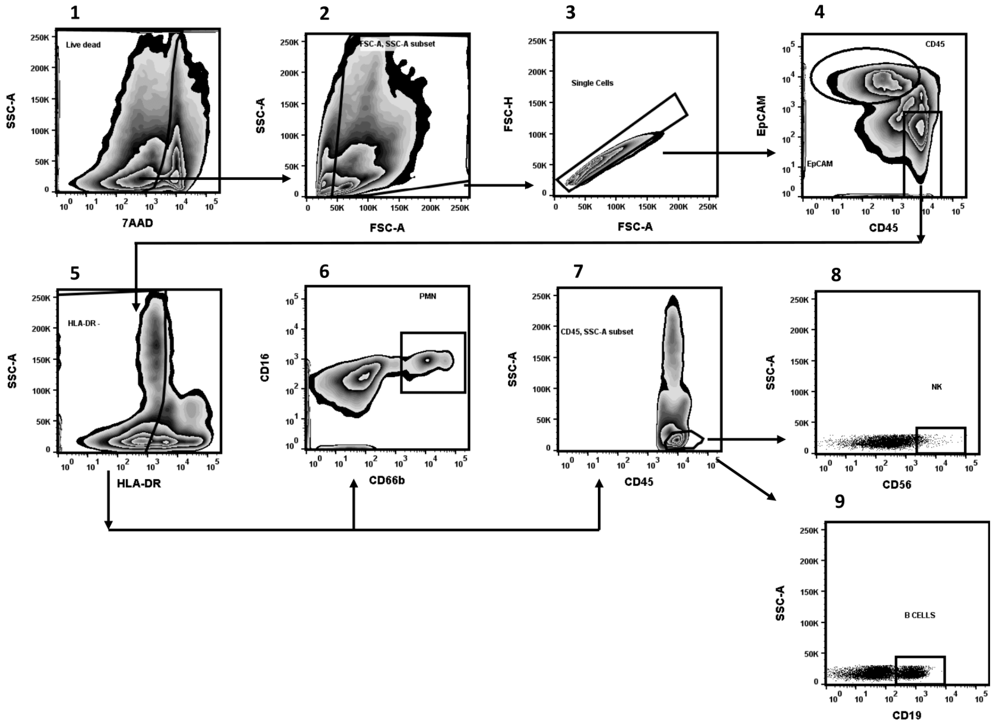

Figure S2. Representative gating strategy for the identification of B lymphocytes, granulocytes, and NK lymphocytes subsets in colon mucosae. In step 1–3 we sequentially excluded dead cells (1), debris (2) and doublets (3). Then, we identified CD45+/EpCam- cells and CD45-/EpCam+ cells (4). Among CD45+/HLA-DR- cells (5) we evaluated granulocytes as CD66b+/CD16+ cells (6). Subsequently, from SSC-A low CD45+ cells (7), we identified NK cells (8) and B lymphocytes (9) as CD56+ and CD19+ cells, respectively.

(colon)

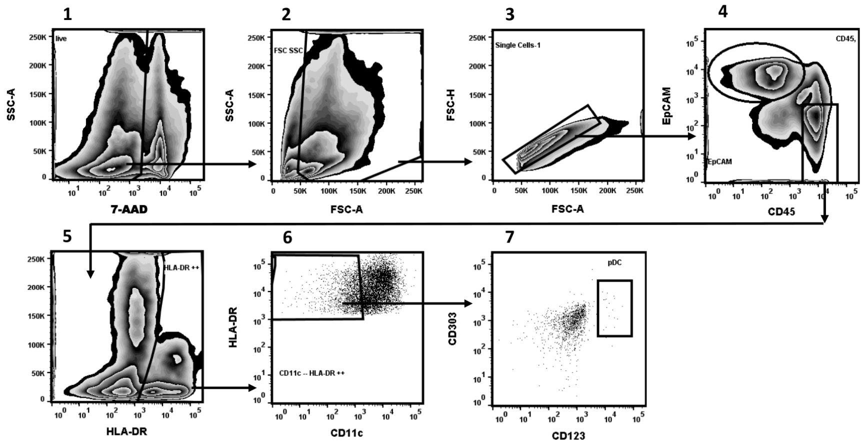

Figure S3. Representative gating strategy for the identification of pDC subset in colon mucosae. In step 1-3 we sequentially excluded dead cells (1), debris (2) and doublets (3). Then, we identified CD45+/EpCam- cells and CD45-/EpCam+ cells (4). Subsequently, we identified CD45+/HLA-DR+ cells (5) and considered only CD11c-/HLA-DR+ cells, excluding the myeloid subset (6). pDCs were gated as CD123+/CD303+ cells (7).

(colon)

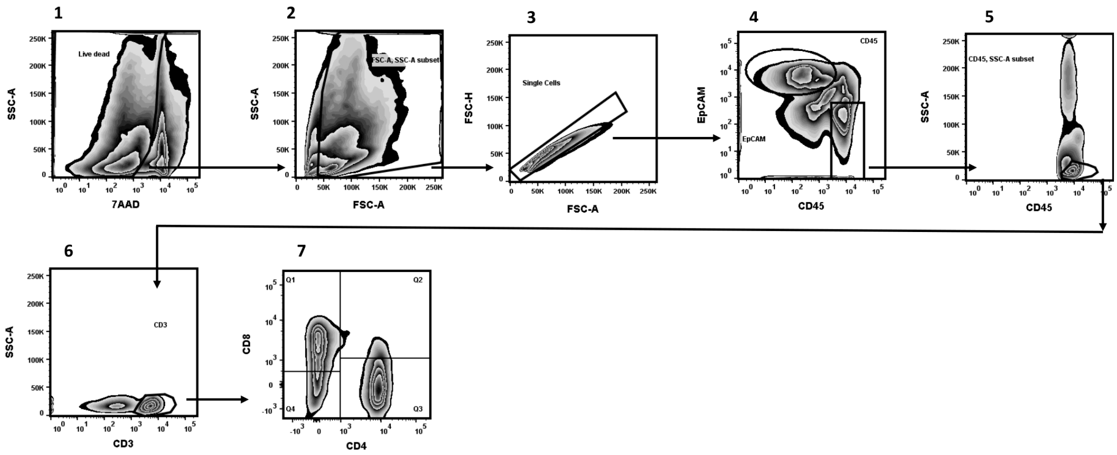

Figure S4. Representative gating strategy for the identification of T lymphocytes subset in colon mucosae. After dead cells, debris and doublets exclusion (step 1–3), we identified CD45+/EpCam- cells and CD45-/EpCam+ cells (4). Subsequentely, T lymphocytes were gated as CD45+/CD3+ with low SSC-A scatter (5 and 6). From CD3+ cells, we evaluated CD4+ or CD8+ T lymphocytes subsets (7).

(colon)

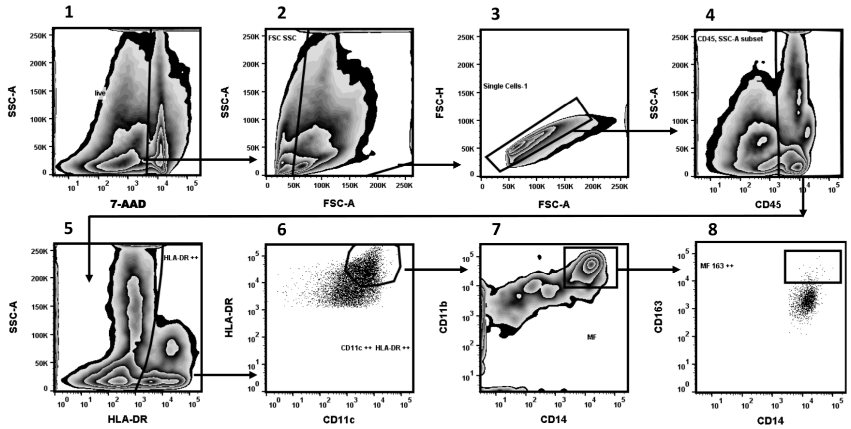

Figure S5. Representative gating strategy for the identification of macrophage subset in colon mucosae. After dead cells, debris and doublets exclusion (step 1–3), we identified CD45+ cells (4) and HLA-DR+ cells (5). We evaluated myeloid subset as CD11c+/HLA-DR+ cells (6). Monocyte/macrophages subset was gated as CD11b+/CD14+ cells (7). Macrophages were evaluated as CD14+/CD163++ cells (8).

(colon)

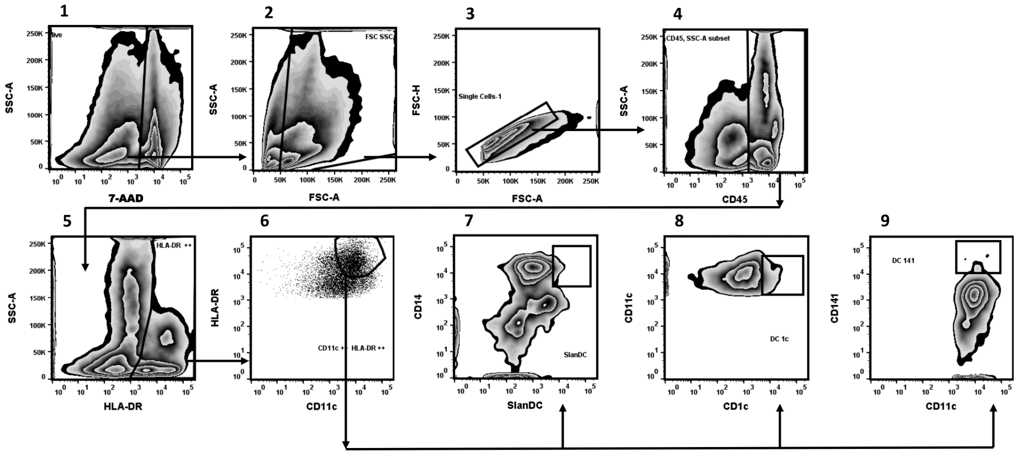

Figure S6. Representative gating strategy for the identification of myeloid cell subsets in colon mucosae. After dead cells, debris and doublets exclusion (step 1–3), we identified CD45+ cells (4) and HLA-DR+ cells (5). We evaluated myeloid subset as CD11c+/HLA-DR+ cells (6). Slan-DC subset were gated as Slan+ and CD14++ bright cells (7). CD1c/BDCA-1 and CD141/BDCA-3 dendritic cells were gated as CD11c+/CD1c+ cells (8) and CD11c+ /CD141++ cells (9), respectively.

(tonsil)

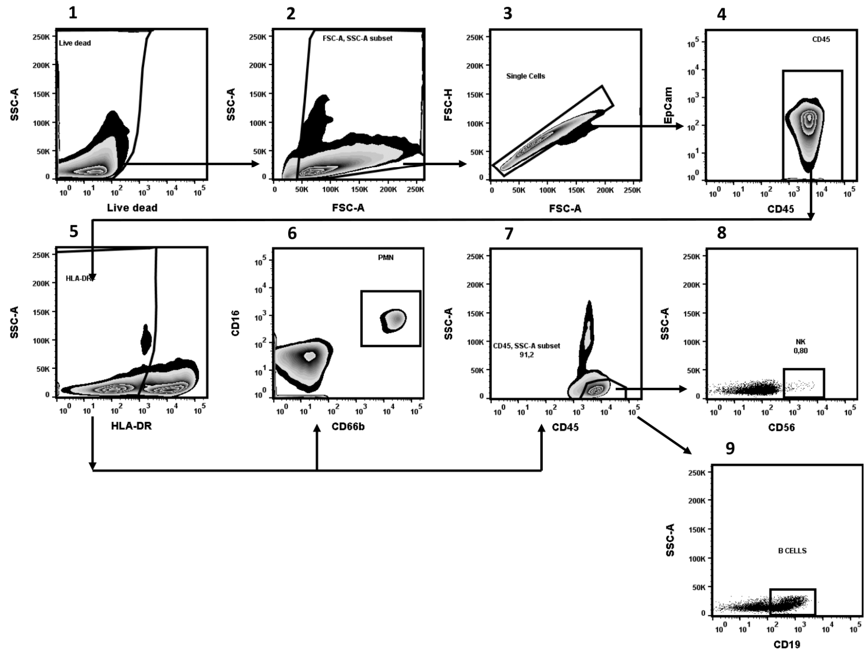

Figure S7. Representative gating strategy for the identification of granulocytes, B lymphocytes and NK cells subsets in tonsils. In step 1-3 we sequentially excluded dead cells (1), debris (2) and doublets (3). Then, we identified CD45+/EpCam- cells (4). Among CD45+/HLA-DR- cells (5), granulocytes were gated as CD66b+/CD16+ cells (6). From SSC-A low CD45+ cells (7), we identified NK cells (8) and B lymphocytes (9) as CD56+ and CD19+ cells, respectively.

(tonsil)

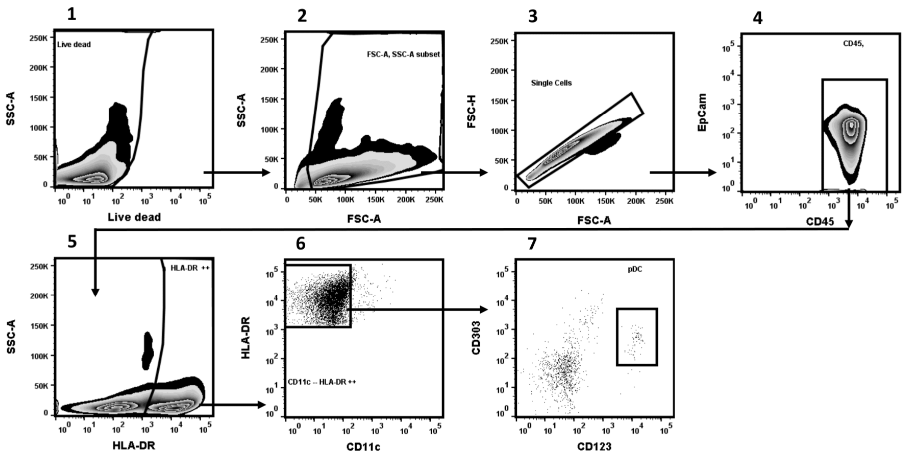

Figure S8. Representative gating strategy for the identification of pDC subset in tonsil. In step 1–3 we sequentially excluded dead cells (1), debris (2) and doublets (3). Then, we identified CD45+/ EpCam- cells (4). Among CD45+/HLA-DR+ cells (5), we considered only CD11c-/HLA-DR+ cells (6).pDCs were gated as CD123+/CD303+ cells (7).

(tonsil)

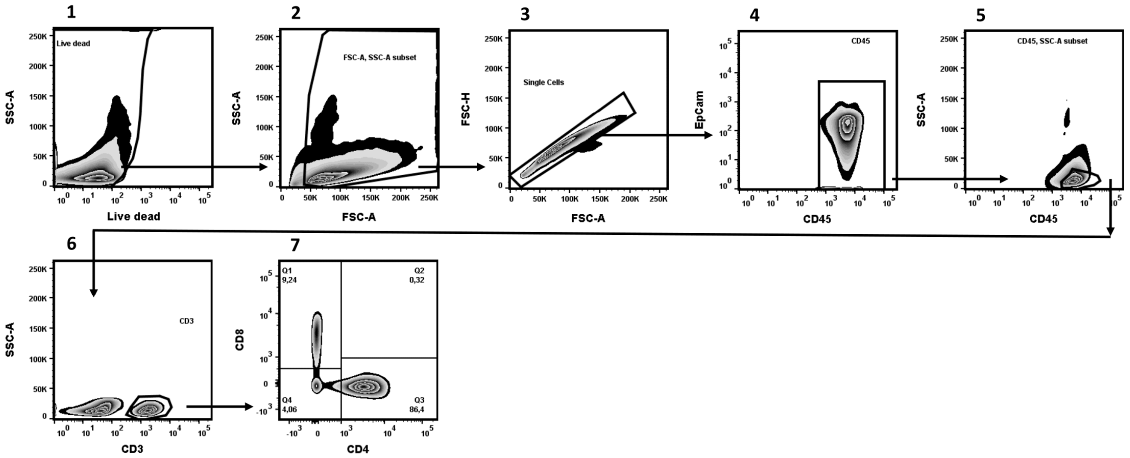

Figure S9. Representative gating strategy for the identification of T lymphocytes subset in tonsil. After dead cells, debris and doublets exclusion (step 1–3), we identified CD45+/EpCam- cells (4). Subsequentely, T lymphocytes were gated as CD45+/CD3+ with low SSC-A scatter (5 and 6). From CD3+ cells, we evaluated CD4+ or CD8+ T lymphocytes subsets (7).

(tonsil)

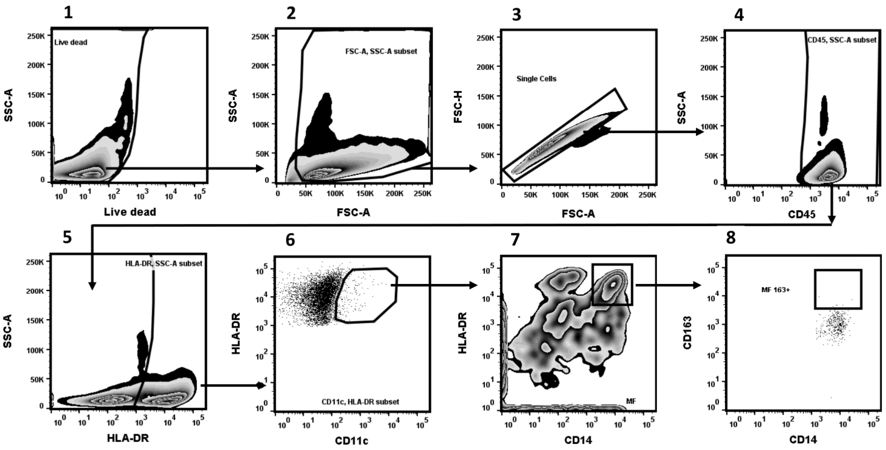

Figure S10. Representative gating strategy for the identification of macrophage subset in tonsil. After dead cells, debris and doublets exclusion (step 1–3), we identified CD45+ cells (4) and HLA-DR+ cells (5). Among CD45+/HLA-DR+ subset, we evaluated myeloid subset as CD11c+/HLA-DR+ cells (6). Monocyte/macrophages subset was evaluated as CD11b+/CD14+ cells (7). Macrophages were gated as CD14+/CD163++ cells (8).

(tonsil)

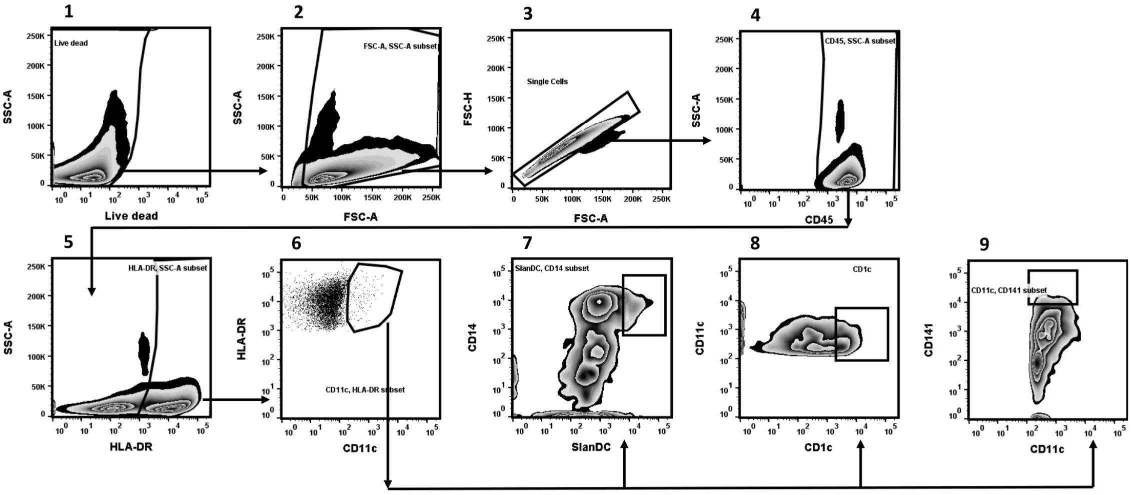

Figure S11. Representative gating strategy for the identification of myeloid cell subsets in tonsil. After dead cells, debris and doublets exclusion (step 1–3), we identified CD45+ cells (4), and HLA-DR+ cells (5). We evaluated myeloid subset as CD11c+/HLA-DR+ cells (6). Slan-DC subset were gated as Slan+ and CD14++ bright cells (7). CD1c/BDCA-1 and CD141/BDCA-3 dendritic cells were gated as CD11c+/ CD1c+ ce [illegible] CD11c+/CD141++ cells (9).

(below PBMC digested VS PBMC undigested, under)

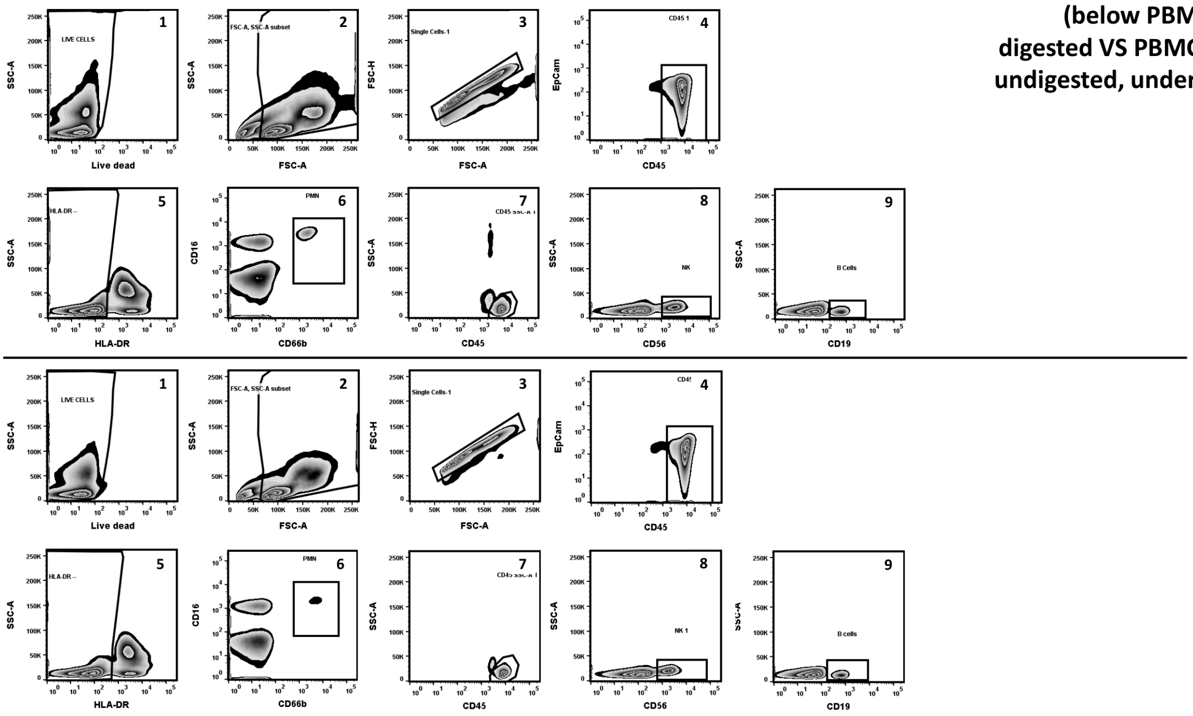

Figure S12. Immunophenotype of B lymphocytes, granulocytes and NK lymphocytes from enzymatically digested PBMCs in comparison with undigested PBMCs. Representative gating strategy for the identification of B lymphocytes, granulocytes and NK lymphocytes in PBMCs shows that there is not any difference between enzymatically treated (upper panel), like colon mucosae, and undigested PBMCs (lower panel). The gating strategies are the same to those described for mucosa (figure S2) and tonsil (figure S7).

(below PBMC digested VS PBMC undigested, under)

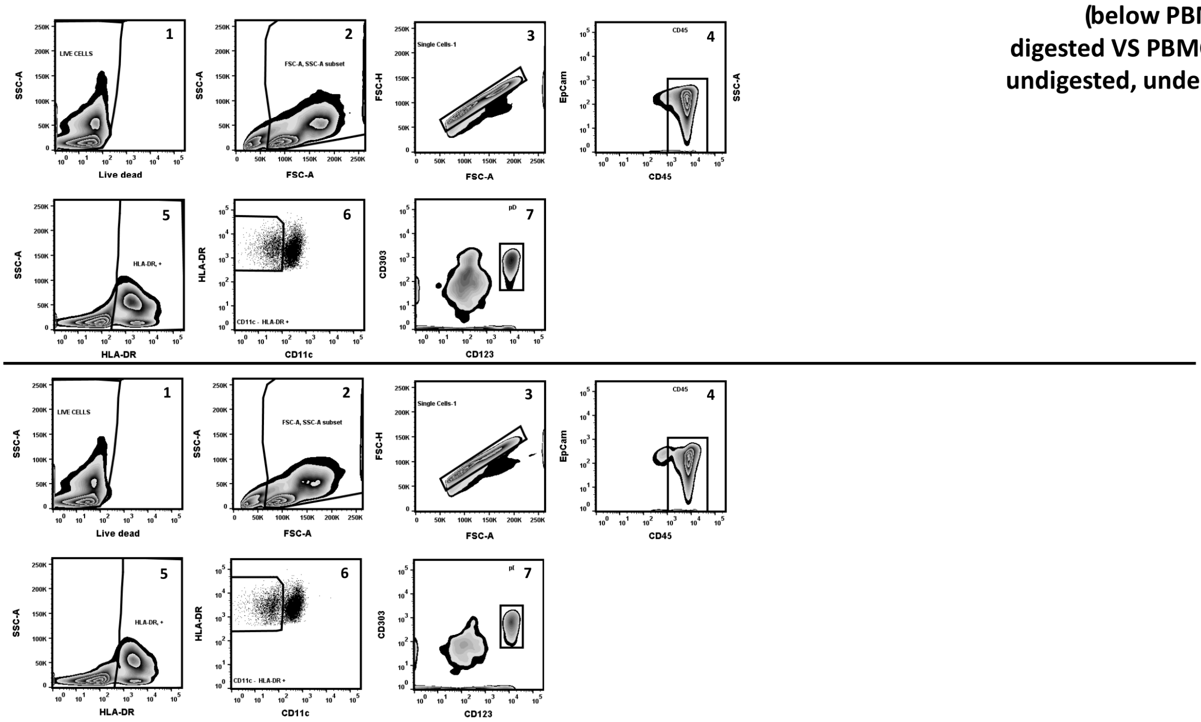

Figure S13. Immunophenotype of pDCs from enzymatically digested PBMCs in comparison with undigested PBMCs. Representative gating strategy for the identification of pDC subset in PBMCs shows that there is not any difference between enzymatically treated (upper panel), like colon mucosae, and undigested PBMCs (lower panel). The gating strategies are the same to those described for mucosa (figure S3) and tonsil (figure S8).

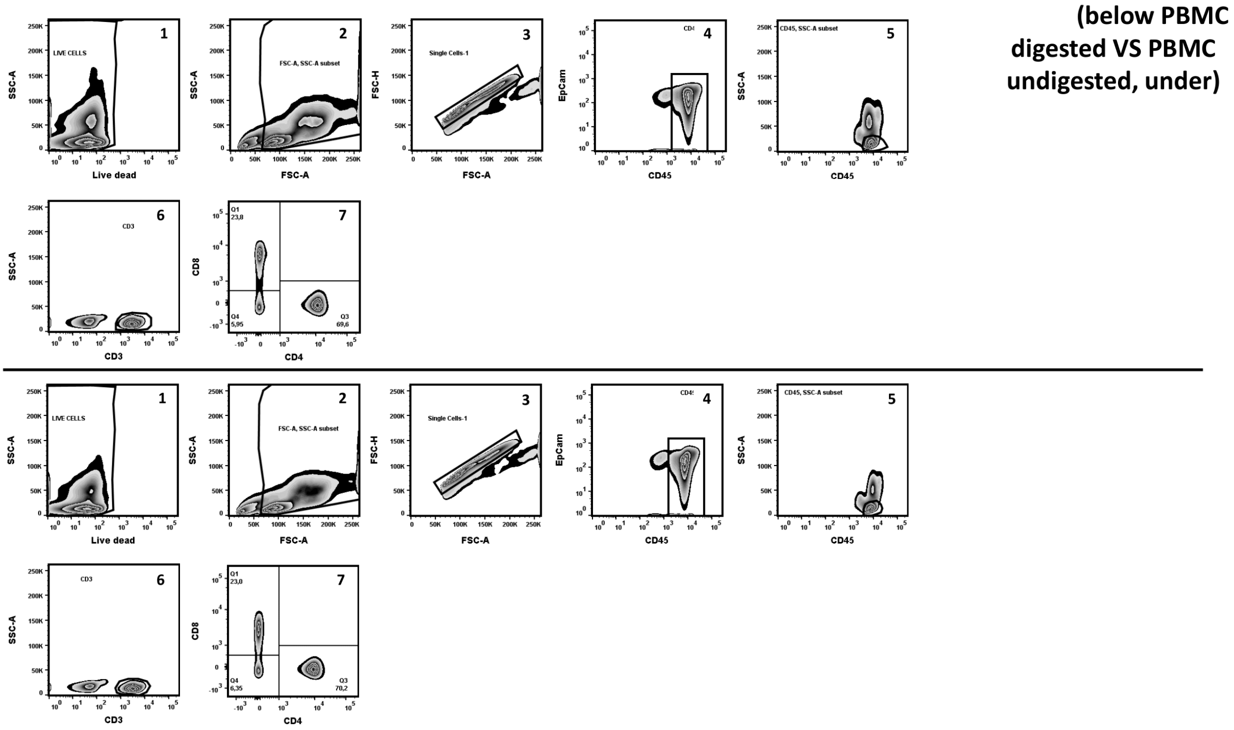

Figure S14. Immunophenotype of T lymphocytes from enzymatically digested PBMCs in comparison with undigested PBMCs. Representative gating strategy for the identification of T lymphocytes subset shows that there is not any difference between enzymatically treated (upper panel), like colon mucosae, and undigested PBMCs (lower panel). The gating strategies are the same to those described for mucosa (figure S4) and tonsil (figure S9).

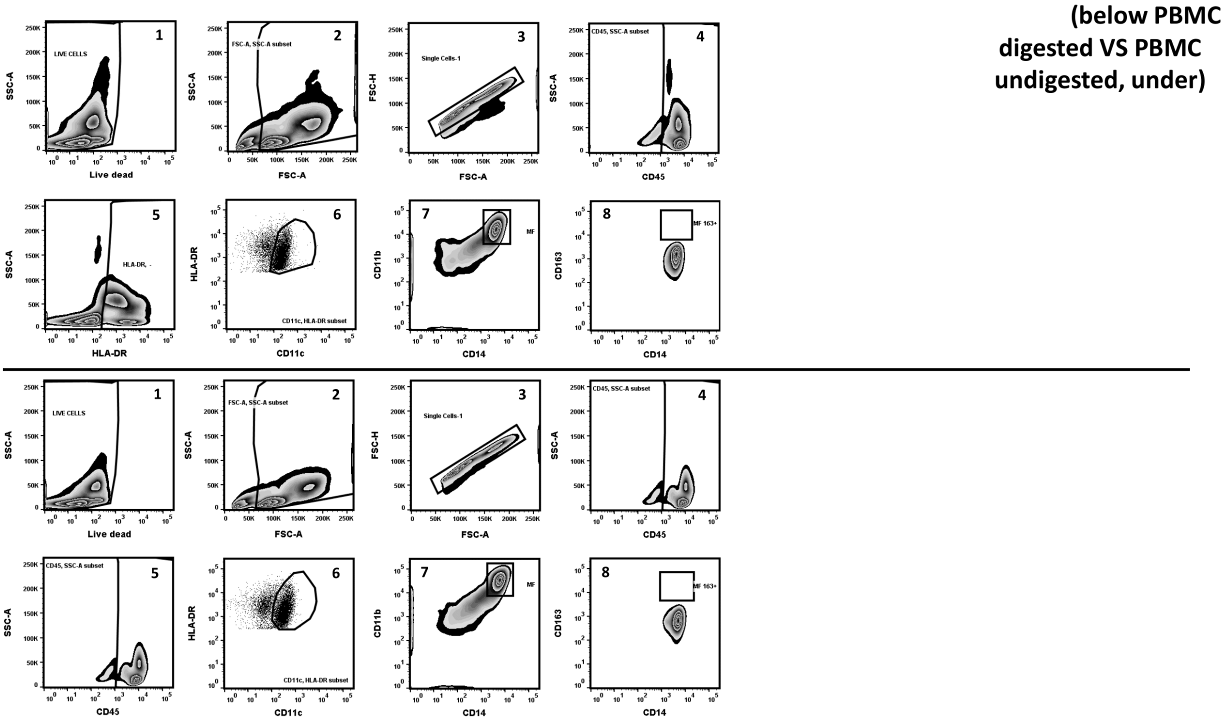

Figure S15. Immunophenotype of macrophages from enzymatically digested PBMCs in comparison with undigested PBMCs. Representative gating strategy for the identification of macrophage subset shows that there is not any difference between enzymatically treated (upper panel), like colon mucosae, and undigested PBMCs (lower panel). The gating strategies are the same to those described for mucosa (figure S5) and tonsil (figure S10).

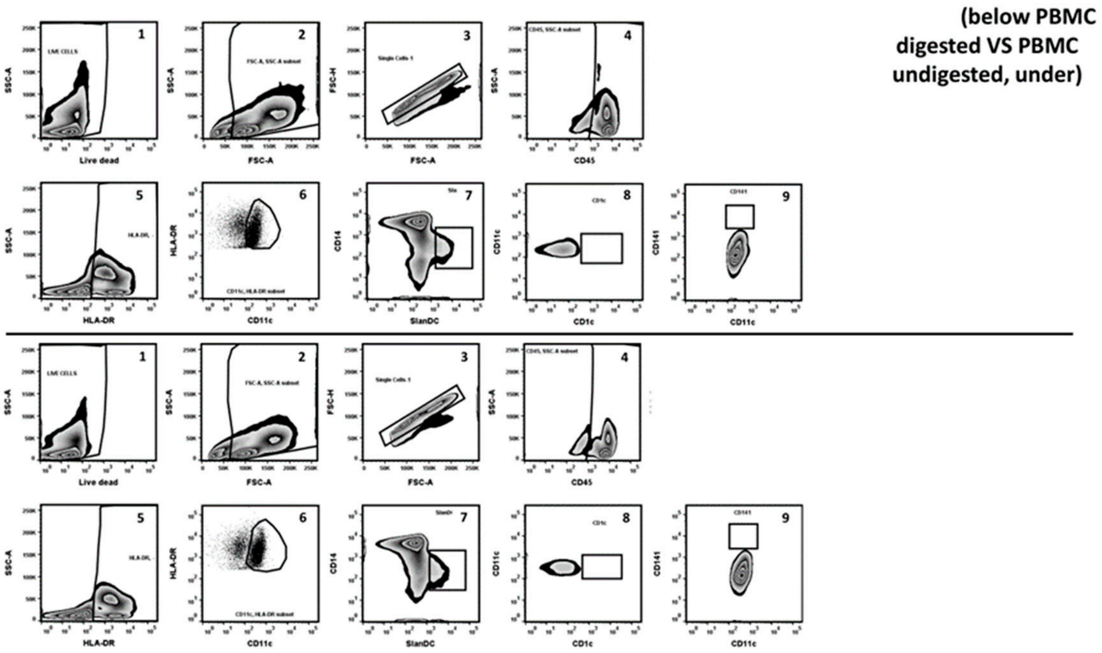

Figure S16. Immunophenotype of myeloid cells from enzymatically digested PBMCs in comparison with undigested PBMCs. Representative gating strategy for the identification of myeloid cell subsets shows that there is not any difference between enzymatically treated (upper panel), like colon mucosae, and undigested PBMCs (lower panel). The gating strategies are the same to those described for mucosa (figure S6) and tonsil (figure S11).

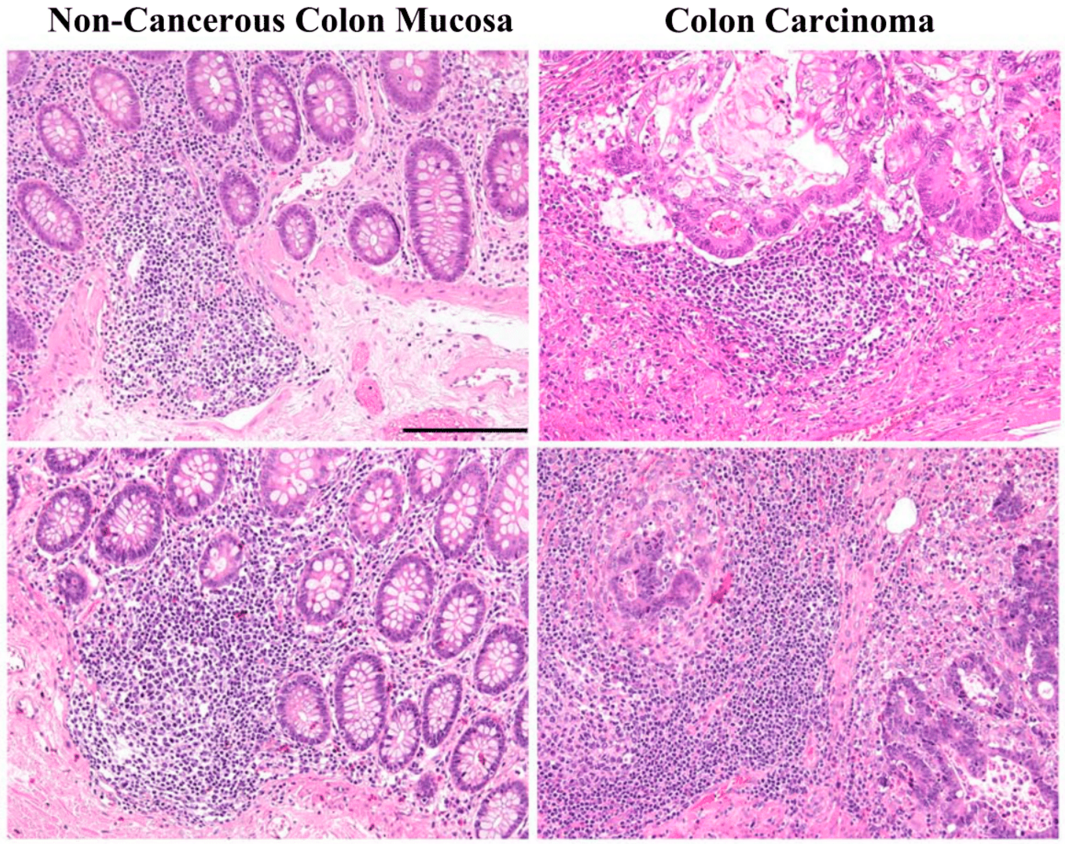

Figure S17. Histology of the Peyer's Patches (PP) in non-cancerous colon mucosa and Tertiary Lymphoid Structures (TLS) in CRC tumor tissues. Two representative H&E showing PP (left panels) and TLS (right panels). Peyer's patches are found in normal colonic mucosa (left panels) and are identified as regular lymphoid aggregates interacting with the normal surface epithelium. In colorectal cancer, TLS correspond to lymphoid structures, mostly irregular in shape, and in close proximity to cancerous tissue. Magnification 100×. Scale bar: 200 μm.
