# Supplementary material for: Immuno-Contexture and Immune Checkpoint Molecule Expression in Mismatch Repair Proficient Colorectal Carcinoma
Source: Cancers (Basel). 2023 Jun 7;15(12):3097. doi: 10.3390/cancers15123097 (PMC10296282; doi:10.3390/cancers15123097)
Supplement: Supplementary file 1 [file cancers-15-03097-s001.zip › cancers-2409922-Supplementary Table S1.pdf]

Supplementary Table S1. List of antibodies used for IHC staining.

Antibody	Clone	Dilution	Source
TCR delta	H-14	1:50	Santa Cruz Biotechnology, Santa Cruz, CA, USA
CD3	LN10	1:70	Leica Biosystems, Wetzlar, Germany
CD4	4B12	1:50	Leica Biosystems
CD8	C8/144B	1:50	Agilent Technologies, Santa Clara, CA, USA
CD163	10D6	1:50	Diagnostic BioSystems, Pleasanton, CA, USA
PD-L1	22C3	1:40	Roche, Basel, Switzerland
TREM2	D8I4C	1:100	Cell Signaling Technology, Danvers, MA, USA
